# The Glyoxalase System in Age-Related Diseases: Nutritional Intervention as Anti-Ageing Strategy

**DOI:** 10.3390/cells10081852

**Published:** 2021-07-22

**Authors:** Gemma Aragonès, Sheldon Rowan, Sarah G. Francisco, Elizabeth A. Whitcomb, Wenxin Yang, Giuliana Perini-Villanueva, Casper G. Schalkwijk, Allen Taylor, Eloy Bejarano

**Affiliations:** 1 Laboratory for Nutrition and Vision Research, USDA Human Nutrition Research Center on Aging, Tufts University, Boston, MA 02155, USA; Gemma.Aragones@tufts.edu (G.A.); Sheldon.Rowan@tufts.edu (S.R.); Sarah.Francisco@tufts.edu (S.G.F.); elizabeth.whitcomb@tufts.edu (E.A.W.); wenxiny7@brandeis.edu (W.Y.); Giuliana.Perini@tufts.edu (G.P.-V.); 2 Department of Ophthalmology, Tufts University School of Medicine, Boston, MA 02155, USA; 3 Friedman School of Nutrition and Science Policy, Tufts University, Boston, MA 02155, USA; 4 Department of Internal Medicine, CARIM School for Cardiovascular Diseases, Maastricht University Medical Centre, 6200 MD Maastricht, The Netherlands; c.schalkwijk@maastrichtuniversity.nl; 5 School of Health Sciences and Veterinary School, Universidad Cardenal Herrera-CEU, CEU Universities, Moncada, 46113 Valencia, Spain

**Keywords:** glycative stress, glyoxalase system, aging, proteotoxicity

## Abstract

The glyoxalase system is critical for the detoxification of advanced glycation end-products (AGEs). AGEs are toxic compounds resulting from the non-enzymatic modification of biomolecules by sugars or their metabolites through a process called glycation. AGEs have adverse effects on many tissues, playing a pathogenic role in the progression of molecular and cellular aging. Due to the age-related decline in different anti-AGE mechanisms, including detoxifying mechanisms and proteolytic capacities, glycated biomolecules are accumulated during normal aging in our body in a tissue-dependent manner. Viewed in this way, anti-AGE detoxifying systems are proposed as therapeutic targets to fight pathological dysfunction associated with AGE accumulation and cytotoxicity. Here, we summarize the current state of knowledge related to the protective mechanisms against glycative stress, with a special emphasis on the glyoxalase system as the primary mechanism for detoxifying the reactive intermediates of glycation. This review focuses on glyoxalase 1 (GLO1), the first enzyme of the glyoxalase system, and the rate-limiting enzyme of this catalytic process. Although GLO1 is ubiquitously expressed, protein levels and activities are regulated in a tissue-dependent manner. We provide a comparative analysis of GLO1 protein in different tissues. Our findings indicate a role for the glyoxalase system in homeostasis in the eye retina, a highly oxygenated tissue with rapid protein turnover. We also describe modulation of the glyoxalase system as a therapeutic target to delay the development of age-related diseases and summarize the literature that describes the current knowledge about nutritional compounds with properties to modulate the glyoxalase system.

## 1. Introduction: Glycative Stress and Unhealthy Aging

**Figure 1:**
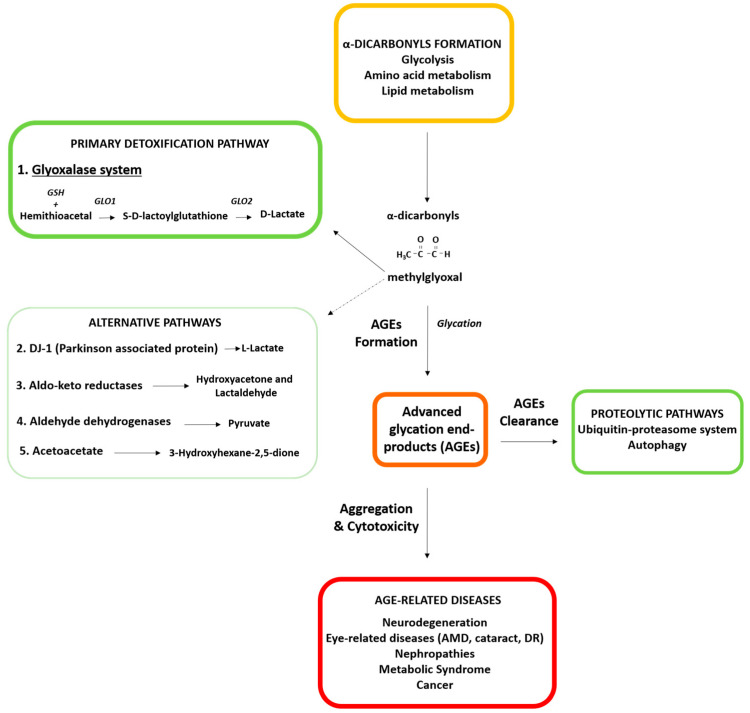
Schematic diagram of α-dicarbonyls formation and detoxification pathways against AGE-derived damage in aging. The formation of highly reactive α-dicarbonyls such as methylglyoxal (MG) is through non-enzymatic degradation of the glycolytic intermediates, including dihydroxyacetone phosphate and glyceraldehyde 3-phosphate and other sources, including aminoacid and lipid metabolism. In order to avoid AGE damage, the glyoxalase system is a primary mechanism that limits the synthesis of AGEs, converting high reactive biomolecules, such as MG, into less reactive biomolecules (D-lactate). This process involves the sequential activity of two enzymes GLO1 and GLO2 and the reduced form of gluthatione (GSH). Other detoxifying mechanisms imply the activity of DJ-1, aldehyde dehydrogenases (ALDHs), aldo-keto reductases (AKRs), and acetoacetate degradation enzymes. Once formed, AGEs can be cleared by two proteolytic pathways: the ubiquitin-proteasome (UPS) system and autophagy. These protective mechanisms (highlighted in green) decline under aging and contribute to the onset of age-related diseases such as neurodegeneration, eye-related diseases (AMD, cataract, DR), nephropathies, metabolic syndrome, and cancer. GLO1: glyoxalase 1; GLO2: glyoxalase 2; GSH: glutathione.

A growing body of literature indicates that accumulation of damaged proteins is a specific hallmark of aging and many age-related diseases, including type 2 diabetes, cancer, neurodegenerative, cardiovascular, and eye-related disorders [1,2,3,4,5,6,7]. Aberrant proteins impair cellular homeostasis by forming non-functional and toxic aggregates and this leads to the inactivation of not only the aberrant protein but can also impair the function of other essential proteins due to the stress on—or insufficiency of—the protein quality control machinery in the cell. One prominent mechanism that leads to aberrant molecules is modification by advanced glycation end-products (AGEs). Dicarbonyl compounds are generated from different metabolic pathways (Figure 1) that involve dietary sugar and carbohydrate metabolism to form AGEs. These dicarbonyl compounds interact with biomolecules, such as proteins, lipids and nucleic acids in a non-enzymatic post-translational modification called glycation. The major glycating dicarbonyls agents are methylglyoxal (MG), glyoxal, or 3-deoxyglucosone [8]. These dicarbonyls are maintained at low levels in homeostatic conditions, but the aging process increases these glycating reagents to pathological levels, enhancing the formation of toxic AGEs and, ultimately, compromising tissue fitness. Given that the formation of AGEs is dependent on glucose concentration, the consumption of high glycemic diets or diabetic conditions lead to a dramatic systemic accumulation of AGEs. This directly correlates with altered metabolism, increased inflammation, and the progression of severe medical conditions. Conversely, the intake of low glycemic diets limits AGE accumulation and is associated with the slower progression of some of these diseases [9,10,11,12,13]. In this context, hyperglycemia imposes additional stress to the age-associated production of glycated proteins and exacerbates the detrimental consequences of AGEs deposits on organ function.

Excessive glycative stress promotes protein insolubility, deregulating signaling and protein quality control pathways. AGEs-derived changes in the proteome perturb signaling pathways in tissue physiology (MAP/ERK, JAK-STAT and PI3K-AKT pathways) that lead to the nuclear translocation of transcription factors involved in multiple cellular functions, including inflammation, apoptosis, ER stress, autophagy, oxidative stress, mitochondrial function, etc. (reviewed in [2,14]). Glycated proteins may also overtax or limit the function of the proteolytic capacities. These changes ultimately contribute to the onset of multiple age-related disorders.

Multiple studies have demonstrated that the formation of MG and MG-derived AGEs is an important factor in the pathogenesis of diabetes and its complications, such as retinopathy, nephropathy, and neuropathy [15,16,17,18,19]. Dicarbonyl stress is also a contributing mediator of obesity and cardiovascular disease [20,21]. MG may contribute to atherosclerosis through several mechanisms, including the accumulation of MG-derived AGEs in atherosclerotic plaques [22] and MG-induced low-density lipoprotein glycation [23]. The association between MG and hypertension has also been observed in several studies, showing increased MG levels in the aorta and kidney tissues [24,25]. Several studies have also confirmed that the accumulation of AGEs is correlated with many neurodegenerative disorders, thus affecting brain function, such as Alzheimer’s disease, Parkinson’s disease, and schizophrenia [26,27,28]. One of the best examples of the relation between AGE accumulation and aging-related consequences occurs in eye tissues resulting in glycation-induced ocular tissue disorders, such as cataract, age-related macular degeneration (AMD) and diabetic retinopathy (DR) [29,30,31]. Regarding cataract, the leading cause of blindness worldwide, lens crystallins become progressively yellow-brown pigmented with age as a result of the accumulation of AGEs byproducts [31]. As well as lens, retinal AGEs increase with age and diabetes, especially in the outer retina. AMD is the leading cause of blindness in older individuals in developed nations. Higher AGE levels are found in AMD patients compared to control subjects as well as in AMD mouse models [32,33,34,35,36]. DR is characterized by the accumulation of AGEs in the retina, inducing microvascular damage [37]. These pathological changes result in irreversible damage to the blood–retinal barrier, macular edema, ultimately resulting in vision loss. In summary, AGEs accumulate throughout the body upon aging and, particularly, in diabetic patients. This compromises organismal homeostasis and contributes to the onset and progress of a plethora of age-related diseases.

There are multiple systems to detoxify AGEs. These include the glyoxalase system, the best characterized mechanism to inhibit the formation of AGEs and one of the routes able to detoxify intermediates of glycation. However, the anti-AGEs capacities decline with age leading to the accelerated accumulation of AGEs in ‘normal’ older tissues. Although there are different defense mechanisms to limit the accumulation of AGEs in tissues, developing them to prevent the accumulation of AGEs and associated pathologies is still not being exploited [38]. In the next section, we summarize the current literature about detoxifying mechanisms focusing on the glyoxalase system to reduce the accumulation of these toxic byproducts in cells and tissues. Finally, we discuss the usefulness of nutritional interventions to boost the glyoxalase system as an anti-ageing strategy.

## 2. Detoxifying Mechanisms against Glycative Stress: Major Role of the Glyoxalase System

Multiple detoxifying mechanisms against the accumulation of AGEs have been reported. Figure 1 is a schematic overview of α-dicarbonyl formation and the different detoxification routes against AGEs-derived damage in aging. The major routes of AGEs synthesis involve the reaction of reactive dicarbonyls derived mainly from the glucose metabolism with primary amines (N-terminal or lysine side chain) or the guanidine group of the arginine side chain [39]. The formation of highly reactive α-dicarbonyls, such as MG, is through the metabolism of the glycolytic intermediates, such as dihydroxyacetone phosphate and glyceraldehyde 3-phosphate, and other sources, including amino acid and lipid metabolism.

AGEs are irreversible and, once formed, can only be eliminated by proteolytic pathways [5,9,40,41]. Two major proteolytic capacities are suggested to contribute to the clearance of AGEs: the ubiquitin proteasome system (UPS) and the autophagic lysosomal proteolytic system (ALPS) [5,9,40,41] (Figure 1). The UPS operates mainly on soluble misfolded proteins. In the UPS, substrates are recognized and tagged with ubiquitin and targeted to the proteasome for degradation. The ALPS consists of the targeting of cargo to the lysosomal compartment for degradation. Autophagic cargo can be diverse, including insoluble proteins, proteinaceous aggregates, and even whole organelles. Both proteolytic pathways are functionally cooperative and increasing literature supports a crosstalk between the two pathways with reciprocal direct and indirect interactions [42,43,44,45,46]. This crosstalk guarantees a backup mechanism and, in the case of the deficiency of one of the routes, the other proteolytic pathway tends to compensate to maintain a proper and functional proteome [47].

Age-related changes in rates of protein degradation were documented for many tissues more than 3 decades ago, even before the molecular characterization of proteolytic pathways was defined [48]. Nowadays, the molecular and cellular decline of the two major proteolytic routes with age is better understood and there are differences in the degrees of decrease between the UPS and lysosomal system. Many reports have shown a tissue-dependent decline in UPS, while autophagic decline seems to be universal (reviewed in [49,50,51]). Regarding autophagy, both lysosomal and autophagosomal compartments undergo striking modifications. Changes that contribute to the malfunction of autophagy include a decrease in lysosomal stability, hydrolase activity, accumulation of indigestible material (lipofusin) in the lysosomal lumen, dysfunctional lysosomal pH, decreased transcriptional level of autophagy-related proteins, decreased stability of the chaperone-mediated autophagy receptor LAMP2A in the lysosomal membrane and decreased association of motor proteins in the autophagic compartments ([49,51,52]). In contrast to autophagy, it is now accepted that changes in proteasome proteolytic abilities with age seem to be more qualitative than quantitative. Changes in the composition of the proteasomal core catalytic activities and modulatory subunits, decreased proteasome expression, as well as changes in the oxidation state of the proteasome subunits and proteasome substrates, contribute to the age-related inhibition of the UPS capacity (reviewed in [53,54]). In some cases, there may just be insufficient capacity of the proteolytic systems to handle to load. Unfortunately, the efficacy of these two mechanisms declines with age, resulting in insufficient capacity to recognize and remove damaged proteins and, therefore, the intracellular accumulation of protein aggregates and dysfunctional organelles [55,56].

Net AGEs levels are determined by the balance of the rate of synthesis or formation and rate of removal. The immediate consequence of the decline in proteolytic capacity is the accumulation of long-lived proteins in aged organisms, many of which accumulate glycation-derived damage in their aminoacid sequences. The accumulation of AGEs occurs in an age-related and -dependent manner ([4,9]) and a recent proteomic analysis in aging research has revealed that AGE biology contains an enriched metabolic pathway associated with age-associated proteomes [57].

Although UPS and ALPS decline with age, there are different protective pathways with the capacity to reduce the synthesis of AGEs. In this review, we focus on these protective mechanisms limiting the biogenesis of AGEs, with an especial emphasis on the glyoxalase system, the primary route for detoxifying reactive dicarbonyls [58]. In this section we will describe the glyoxalase system in detail. We also briefly describe other mechanisms in the detoxification of AGEs: Parkinson-associated protein DJ-1, aldehyde dehydrogenases (ALDHs), aldo-keto reductases (AKRs), and acetoacetate degradation.

### 2.1. Glyoxalase System: The Major Detoxifying Route for Reactive Dicarbonyls

A vast literature supports the glyoxalase system as the major detoxifying route for reactive dicarbonyls in the cytosol of all mammalian cells [58]. The glyoxalase system is the best-characterized pathway for the metabolism of MG. Genes for glyoxalases are evolutionarily conserved and widely distributed in various living systems, such as humans, plants, yeast, bacterial, fungi, and protists. The presence in many diverse taxa points out the highly importance of glyoxalase enzymes in physiological function of biological life. The combined activities of glyoxalases 1 and 2 (GLO1, GLO2) catalyze the conversion of reactive, acyclic α-oxoaldehydes into the corresponding α-hydroxyacids [58]. These reactions also require catalytic GSH. In the initial step, GLO1 converts its substrate, hemithioacetal, formed by a spontaneous reaction of the aldehyde of the dicarbonyl MG and GSH, into S-D-lactoylglutathione. Then, GLO2 hydrolyzes S-D-lactoylglutathione to D-lactate and reforms GSH (Figure 1). The activity of GLO1 is directly proportional to GSH concentration. The GLO1 activity decreases when GSH is removed, such as upon oxidative stress, when GSH is converted to GSSG [59].

MG is formed during glycolysis and gluconeogenesis by the degradation of dihydroxyacetone phosphate and glyceraldehyde 3-phosphate, as well as by the catabolism of threonine, the oxidation of ketone bodies, and the degradation of glycated proteins. Other substrates, including glyoxal, phenylglyoxal and hydroxypyruvaldehyde, are also metabolized via this pathway [60]. GLO1, the rate-limiting enzyme in the glyoxalase system, catalyzes the primary detoxification step [61], thus the alteration of GLO1 protein is involved in many pathological processes in aging, such as in diabetes, neurodegenerative diseases, cancer, and eye-related diseases [20].

**Figure 2:**
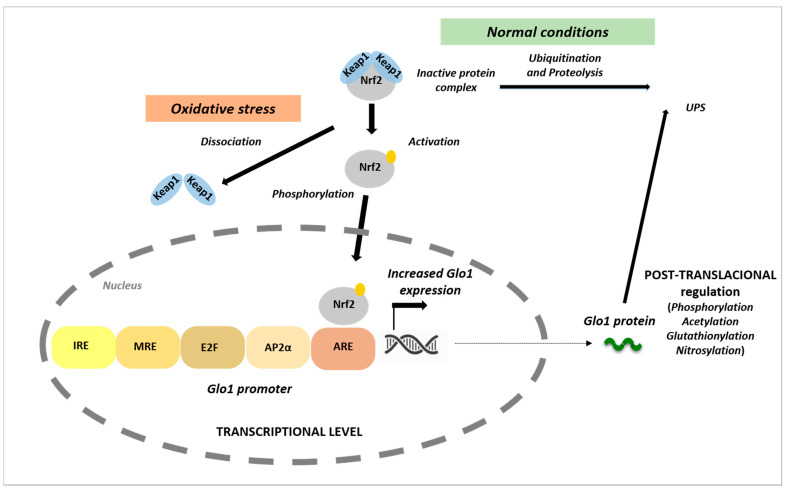
Mechanisms of glyoxalase 1 (GLO1) regulation. GLO1 activity can be regulated through multiple mechanisms, including transcriptional regulation and post-translational modifications. The *Glo1* promoter contains various regulatory elements, such as antioxidant response (ARE), metal-response (MRE) and insulin-response (IRE) elements, and binding sites for AP-2α and E2F. In normal conditions, the nuclear factor erythroid 2-related factor 2 (NRF2) is complexed with KEAP1, a substrate adaptor protein for cullin-3-dependent E2 ubiquitin lipase complex, directing NRF2 for degradation by the ubiquitin proteasome system (UPS). Oxidative stress leads to the destabilization of the complex NRF2-KEAP1, causes the detachment of NRF2 that is translocated to the nucleus where triggers the upregulation of different antioxidants genes. Binding of NRF2 to the *Glo1-*ARE increases expression of GLO1. Under hypoxia conditions Glo1 expression is inversely regulated by hypoxia-inducible factor 1α (HIF1α). Different post-translational modifications in the cytosol can impact GLO1 stability.

The regulation of GLO1 expression and activity is complex and still not well-understood (Figure 2). The *GLO1* promoter sequence contains a metal-response element (MRE), an insulin-response element (IRE), an early gene 2 factor isoform (E2F), an activating enhancer-binding protein 2α (AP-2α), and an antioxidant-response element (ARE). The function of the IRE and MRE were confirmed in reporter assays where insulin and zinc chloride treatment produced an increased transcriptional response [62]. Similar functional activities were observed for E2F and AP-2α [63,64]. The ARE located in exon 1 of *Glo1* serves to join *Glo1* to the nuclear factor erythroid 2-related factor 2 (NRF2) stress-responsive transcriptional system [65]. Several genes related to MG metabolism and protection against oxidative stress are under the control of the NRF2–ARE pathway [66]. NRF2 is complexed with KEAP1, a substrate adaptor protein for cullin-3-dependent E2 ubiquitin lipase complex, directing NRF2 for degradation by the 26S proteasome upon physiological conditions. Oxidative stress leads to the destabilization of this complex, causing the translocation of NRF2 to the nucleus, and triggering the upregulation of antioxidant genes [67,68]. The binding of NRF2 to the Glo1-ARE increases the basal and inducible expression of GLO1. [65]. NRF2 and antioxidant responses are also upregulated when MG causes the dimerization of KEAP, liberating Nrf2 [69].

Several studies show that NRF2 increases GLO1 activity and alleviates intracellular MG stress; thus, the modulation of GLO1 by NRF2 agonists resulted in a decrease in MG and MG-derived protein adducts in both cells and tissues [70,71,72,73]. Moreover, hepatic, brain, heart, kidney and lung *Glo1* mRNA and protein were decreased in NRF2 knockout mice [65]. Altogether, these reports suggest that GLO1 is a downstream target by which the NRF2/KEAP1 pathway performs its protective functions by decreasing MG and dicarbonyl stress. However, the inflammatory activation of the NF-κB (nuclear factor κB) with NRF2 diminishes *Glo1* expression [74]. *Glo1* expression is also negatively regulated by HIF1α (hypoxia-inducible factor 1α) under hypoxic conditions, an important physiological driver of dicarbonyl stress [75].

Along with transcriptional regulation, there is also post-translational regulation of GLO1 protein (Figure 2). GLO1 is acetylated by cytosolic sirtuin-2 [76,77], and its expression may be decreased by activation of RAGE (receptor for advanced glycation end-products); however, these mechanisms are not clearly understood [78]. A recent study showed that GLO1 protein can be modified by the phosphorylation of threonine 107 (T107) and the nitrosylation of cysteine 139 [79]. In this study, the phosphorylation of T107 by calmodulin-dependent kinase II delta in the GLO1 protein was reported as a precise mechanism regulating the glyoxalase system. Specifically, the phosphorylation of GLO1 at T107 affects the kinetic efficiency of MG detoxification and proteasomal degradation rate. Thus, its altered status is associated with the development of age-related diseases [79].

### 2.2. Alternative Detoxification Mechanisms as Putative Backup Systems to Compensate the Lack of Glyoxalase Activity

While the primary mechanism for detoxifying reactive dicarbonyls is the glyoxalase system, there are alternative routes with the capacity to detoxify dicarbonyls formed during sugar metabolism. These include ALDHs, AKRs, the Parkinson associated protein DJ-1, and scavenging by acetoacetate to form 3-hydroxyhexane-2,5-dione (3-HHD) [80]. The physiological relevance of these systems remains unclear and it has been questioned whether or not these enzymes are crucial for the detoxification of AGEs in tissues due to the high activity of the glyoxalase system. They seem to be components of back-up systems that operate in the absence of glyoxalase activity although a tissue-dependent role of these routes cannot be discounted.

DJ-1, also known as Parkinson disease protein 7 (PARK7), plays an essential role in Parkinson disease (PD). The lack of functional DJ-1 protein has been shown to cause autosomal recessive PD [81,82]. DJ-1 was reported to have two different activities: (1) glyoxalase activity in vitro, converting MG into lactate and preventing MG-induced tissue damage in *Caenorhabditis elegans* [83] and (2) deglycase activity in vitro, reducing early stage MG byproducts [84]. Recently, other studies have also showed that DJ-1 plays a relevant role as DNA deglycase [85,86,87]. The detoxifying capacity of DJ-1 in the absence of glutathione (GSH) makes this an alternative route to the glyoxalase system, which requires the presence of GSH. However, Pfaff et al. using both DJ-1 knockdown in *Drosophila* cells and DJ-1 knockout in the whole organism, observed no differences in the accumulation of MG protein adducts [88].

AKRs are a superfamily of proteins able to reduce aldehydes and ketones into primary and secondary alcohols. AKRs metabolize MG to hydroxyacteone or lactaldehyde. Some studies showed that the transgenic expression of both human and mouse aldo-keto reductases in rodent fibroblast cells protects against MG-induced damage, suggesting that AKRs can participate in MG detoxification and a reduction in AGEs levels [89,90,91]. High AKR1B3 activity was detected in *Glo1* knockout mouse Schwann cells as well as increased expression during MG exposure, suggesting that it could be a compensatory mechanism induced by lack of the glyoxalase system or excessive glycative stress [92]. Interestingly, the lack of AKR1B3 resulted in higher levels of MG and AGEs in the hearts of diabetic mice [91].

ALDHs are another group of α-dicarbonyl metabolizing enzymes that oxidize MG to pyruvate. ALDH expression was increased in mouse Schwann wild-type cells upon MG treatment [92]. In a zebrafish model, *glo1* knockout fish showed that induced ALDH activity compensates for the lack of GLO1 [93]. However, at least in mouse, the compensatory mechanisms are tissue-dependent, as the increased expression of AKRs and ALDHs were observed in liver tissue but only AKRs were reported in kidneys in *Glo1* knockout mice [94]. In human studies, the 3-DG metabolite produced by aldehyde dehydrogenase 1A1 (ALDH1A1) activity was increased in plasma and erythrocytes of diabetic patients [92]. Recently, it was also shown that ketone body acetoacetate reduced MG concentration by a non-enzymatic reaction during diabetic and dietary ketosis [95,96]. They found that this metabolic route involves a non-enzymatic aldol-reaction between MG and the ketone body acetoacetate, leading to 3-hydroxyhexane-2,5-dione, which is present in the blood of insulin-starved patients. Alternative pathways that might compensate the deficiency of glyoxalase system could potentially generate toxic molecules such as γ-diketones, which are associated with peripheral axonal degeneration and testicular injury [97,98].

Although there is no systematic aging analysis of proteins involve in GLO1-independent alternative pathways, age-related changes of those molecular players have been reported. For example, there is a correlation between DJ-1 levels of expression and oxidative stress and different reports showed an increase in DJ-1 with age. DJ-1 mRNA and protein levels increased from 8 to 20 weeks of age in mice [99] and DJ-1 levels significantly increased as a function of age in human cerebrospinal fluid [100]. In ocular tissues, it has been shown that the DJ-1 is expressed in retinal pigment epithelium and photoreceptors and the expression increased in old eyes [101]. It might reflect a compensatory mechanism due to the decline in glyoxalase system activity.

### 2.3. Tissue-Dependent Activity of Glyoxalase System

**Figure 3:**
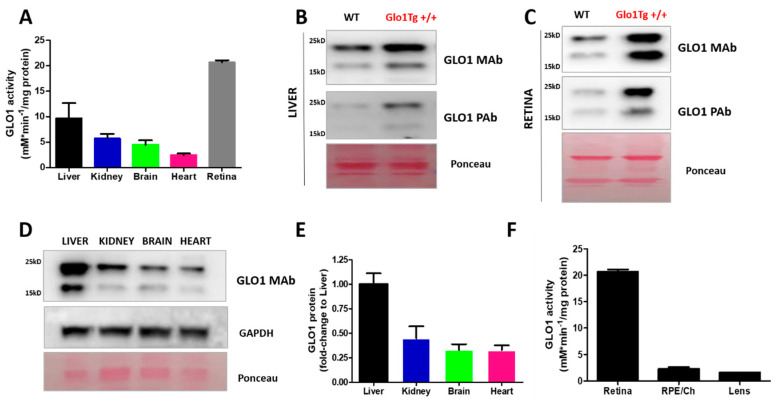
A comparative analysis of GLO1 protein and activity in ocular and non-ocular tissues. (**A**) GLO1 activity was assayed in non-ocular tissues and retinal tissues from WT mice as previously described [29] and activity was expressed as percentage (%) compared to liver. (**B**) Liver and (**C**) retina representative Western blot analysis of WT and *Glo1* overexpression transgenic mice (Glo1 Tg+/+) using a monoclonal antibody (non-commercial) and polyclonal antibody for Glo1 (commercial, GeneTex) [36,103,104]. (**D**) Representative Western blot analysis of non-ocular tissues extracts (50ug) of WT mice using a monoclonal antibody for Glo1 (non-commercial) and (**E**) protein quantification of GLO1 normalized to control loading (Ponceau staining). (**F**) GLO1 activity was performed in ocular tissues (Retina, RPE/Choroid, and Lens) from WT mice as previously described [29] and activity was expressed as milliunits per milligram of protein. Values are mean ± SEM. Sample size is *n* = 4 from the GLO1 protein and activity assays.

Although GLO1 is a ubiquitous protein, the levels of this enzyme are regulated in a tissue-dependent manner. In order to evaluate the role of the glyoxalase system in different tissues, we examined the expression and activity of GLO1 in non-ocular (liver, brain, heart and kidney) and ocular tissues (retina, RPE/choroid and lens) from wild type C57BL/6J mice. Using antibodies that specifically recognize GLO1, Western blotting and immunohistochemistry were performed to quantify protein levels. GLO1 activity in cytosolic extracts was determined spectrophotometrically as the initial rate of formation of S-D-lactoylglutathione, as previously reported [30,102]. These results are summarized in Figure 3.

Previous published data indicated that retina and liver display the highest activity of GLO1 ([30]; Figure 3A). Note that retinal activity was the highest value while that liver, kidney, brain and heart only represented 46%, 27%, 22% and 11% of detoxifying retinal capacity, respectively. We evaluated if the activity of GLO1 correlated with the level of the enzyme by assessment of GLO1 protein levels by Western blotting. The antibody against GLO1 was previously validated in previous reports and used for the analysis of GLO1 in retinal samples [36,103,104]. As a positive control, a comparative analysis was also performed in retina and liver tissues from transgenic mice overexpressing GLO1 on C57BL/6J (B6) background [105]. To examine the levels of GLO1, we used two different antibodies: a polyclonal rabbit antibody (commercial antibody from GeneTex) and a monoclonal mouse antibody (non-commercial antibody) reported in different animal models for the study of GLO1 biology [103,106]. We were able to detect GLO1 protein in liver and retina wild-type tissues by Western blotting, and we found the highest expression in transgenic mice in both tissues (Figure 3B,C and Supplementary Figure S1). Two bands were recognized for both antibodies. The differential electrophoretic profiles of these GLO1-positives suggest that posttranscriptional changes could be vital in the role of the protein. Accordingly, a recent study indicated that phosphorylated GLO1 is more efficient and more stable, supporting these post-transcriptional alterations as a precise mechanism regulating GLO1 activity [79]. However, there is scant information about how post-transcriptional modifications modulate glyoxalase 1 activity.

As expected, we found GLO1 protein in all non-ocular tissues analyzed, with liver showing highest expression. The relative order of GLO1 expression was liver > kidney > brain > heart (Figure 3D,E). This corroborates the results of a previous study [30]. There is limited information about the role of GLO1 in ocular tissues. As we previously reported, the enzymatic assay revealed that GLO1 activity is ~10 fold higher in the retina compared to lens or RPE/choroid (Figure 3F, [30]). The overexpression of glyoxalase I improves human retinal pericyte survival under hyperglycemic conditions [107] and an angiotensin receptor blocker that restore GLO1 in diabetic rats was shown to reduce retinal acellular capillaries [18]. Additionally, the lack of GLO1 in Zebrafish impacts the adult retina vessel architecture, although increased angiogenic sprout formation is only observed in in glo1^–/–^ overfed zebrafish but not in normal feeding [93].

**Figure 4:**
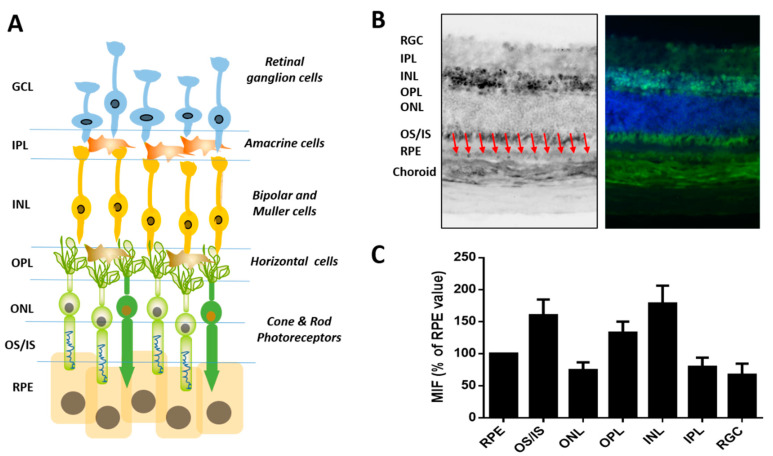
Immunohistochemistry of GLO1 in mouse retinal tissues. (**A**) Cross-sectional, cellular schematic of the retina illustrating its three primary layers comprised of the ganglion cell layer (GCL), containing retinal ganglion cells (RGC), inner nuclear layer (INL), hosting interneurons of amacrine, bipolar and horizontal cells as well as Müller glial cells, and outer nuclear layer (ONL), housing rod and cone photoreceptors. The sensory tissue, or neuroretina, is connected to the retinal-pigmented epithelium (RPE). Red arrows indicated the RPE layer. (**B**) Representative picture of GLO1 immunostaining in retinal samples from WT mice. (**C**) Mean intensity fluorescence of GLO1 normalized to the value in the RPE. Data shown are mean ± standard errors of the means (SEM).

The retina is a highly complex, very dynamic tissue with diverse cell types (Figure 4A). Blood flow, and consequent exposure to xenobiotics and other stressors, is among the highest in the body. Each morning, 10 per cent of outer tips of retina photoreceptors are shed and must be removed by adjacent retinal pigmented epithelial cells. We performed immunohistochemical analysis to characterize for the first time the spatial differences of GLO1 in retina. GLO1 protein was present in all cell types within the retina, with high levels within cell bodies of the inner nuclear layer and ganglion cell layer. Photoreceptor cell bodies in the outer nuclear layer had lower levels. In photoreceptors, most GLO1 protein was found within the inner and outer segments. The RPE also had high levels of GLO1 protein, whereas the choroid and sclera had lower amount of GLO1 protein (Figure 4B,C).

Our results in the retina are relevant because the retina is a highly differentiated postmitotic tissue, where glycation-derived damage cannot be reduced by cellular division [5,9]. Furthermore, changes in GLO1 have been associated with retinal damage [108]. A similar scenario might occur in other tissues composed of cells with low regeneration capacity, such as the central nervous system, where the vast majority of neurons are post-mitotic. The evaluation of GLO1 levels along with cell-specific markers might allow us to evaluate the cell-to-cell variation within a given tissue. Our results suggest that the high level of retinal GLO1 protein and activity might play an important protective role against AGE-derived damaged with age.

## 3. Biology of the Glyoxalase System in Aging and Age-Related Diseases

### 3.1. The Involvement of Glyoxalase System in Aging and Age-Related Diseases

The aging process is characterized by a gradual impairment of the functional properties of cells, tissues and whole organs. This may start with damage to cellular processes, such as mitochondrial function, proteostasis, and detoxifying systems. Among the insults or stresses associated with these compromises are an age-related decline of GLO1 activity, MG-derived AGEs and age-related tissue dysfunction [109]. For example, Morcos et al. found a broad decline of GLO1 expression and activity in *Caenorhabditis elegans* with age and that overexpression of *Glo1* increased median and maximum lifespan [110]. In addition, the loss of *Glo1* decreased lifespan, and demonstrated that the decrease in GLO1 activity increases mitochondrial ROS production, ultimately reducing lifespan. Sharma-Luthra et al. showed diminished GLO1 activity in mouse liver and spleen during the lifespan. However, GLO1 activity was increased in kidney at 24 months [111]. GLO1 activity of rat liver tissue decreases with age, as well as under hypoxia in young rats [112]. GLO1 activity also declined during aging by approximately 50% in liver tissues of 80-week-old wild-type mice, as compared with 10-week-old wild-type mice [79]. This reduction in GLO1 was most pronounced in liver. It was also observed in diabetic mice [79]. Interestingly, the observed decline of GLO1 activity was linked to a loss of GLO1 phosphorylation upon aging. GLO1 phosphorylation was not examined in other tissues. It remains to be clarified if tissue-dependent differences in the ratio of this post-transcriptional modification could be behind differences in GLO1 activity.

In humans, several studies have investigated the impact of aging on GLO1. These studies found a reduction in GLO1 activity in multiple tissues, such as arterial tissues, lens, brain and red blood cells with age [113,114,115,116,117,118,119]. However, a systematic analysis to clarify in vivo changes of GLO1 activity associated with age has not been published to date.

Obesity is a risk factor for chronic diseases during aging and there is increasing evidence supporting a genetic link between *Glo1* and obesity [20,120,121]. A meta-analysis of quantitative trait loci in mice linked *Glo1* to obesity-related phenotypes [122]. In humans, *GLO1* was associated with upper-arm circumference and supra-iliac skinfold thickness, anthropometric measurements used as markers of hypertension [123]. In leptin-deficient ob/ob mice, a mouse overeating model of obesity, GLO1 protein was decreased in the liver [124]. Additionally, increased weight gain in mice lacking *Glo1* fed with high fat diets and decreased weight gain and adiposity in *Glo1* overexpressing transgenic mice with high fat diet have been observed [125,126], supporting a functional role of GLO1 and dicarbonyl stress in obesity. Altogether, this evidence indicates that the downregulation of GLO1 might lead to cell and tissue dysfunction caused by dicarbonyl stress, which is a potential driver of obesity [20,121].

Given that the level of AGEs is dependent of blood glucose concentration, GLO1 expression and activity has also been investigated with regard to diabetes. Some studies showed that the overexpression of *Glo1* in transgenic rats and mice could delay the development of microvascular complications in diabetes, such as nephropathy, retinopathy and neuropathy [104,108,127]. In vitro studies showed that the reduction in GLO1 activity induced an accumulation of MG in endothelial cells under high glucose treatment [61,128,129]. In contrast, overexpression of GLO1 in these cells during glycative stress in vitro delayed the formation of AGEs [61]. Additionally, in vitro analysis showed that GLO1 overexpression reduced vascular complications under hyperglycemic conditions [107]. Several studies in both mouse and rat diabetic models showed that GLO1 protein or activity was decreased in different tissues, including kidney, sciatic nerve, liver and extra-renal tissues [15,16,17,107,130]. On the other hand, red blood cells of diabetic mice had higher GLO1 activity than non-diabetic mice [131]. Regarding human studies, GLO1 activity was increased in red blood cells in diabetic individuals compared to healthy subjects [116]. Additionally, patients with diabetes and microvascular complications had significantly higher activity of GLO1 in red blood cells compared to patients without complications, indicating a potential compensatory response to elevated dicarbonyl stress [116]. Together, these findings are consistent with glucose elevation, eliciting a hormetic effect [80].

All published epidemiologic studies indicate that people who consume lower glycemic index diets are protected against AMD, and even against the progression of AMD [132,133]. Consistent with these data, the more one subscribes to a Mediterranean, lower glycemic index diet, the better the protection against early or late AMD [134]. The converse is also observed. These data imply that there are associations between our ability to process glucose and risk for AMD. They also suggest that protection against excess glucose or glucose metabolites, by GLO1 would be salutary. There is limited literature regarding the role of the glyoxalase system in eye disease. However, increasing evidence indicates a link between the impairment of the glyoxalase system and the development of DR. Expressions of *GLO1* and *GLO2* are downregulated in patients with DR, suggesting that dysfunction in this detoxifying system could be behind the development of retinopathy in humans [135,136]. Interestingly, a polymorphism that disrupts *GLO1* promoter activity has been associated with retinopathy in diabetic subjects [137]. GLO1 activity was increased in retinal extracts from a mouse model that was protected from hyperglycemia-induced vasoregression (a hallmark of DR) by loss of TRPC (Transient Receptor Potential-Canonical) channels [138]. More directly, a transgenic rat model overexpressing GLO1 inhibited retinal AGE formation and prevented DR lesions [108]. Overall, we speculate that boosting GLO1 activity could reduce retinal AGEs in a diabetes context and prevent AGE-related pathologies [30].

There is a robust literature indicating that consuming lower glycemic index diets limits risk for cardiovascular diseases (CVD) [132]. Consistent with these findings, a large cohort study investigating genome-wide gene expression associations found a link between decreased GLO1 and CVD [139]. In haemodialysis patients, the *GLO1* 419A>C polymorphism was associated with increased risk of CVD complications [140]. Further studies showed a high mortality rate in patients with homozygous GLO1 419CC mutation [141]. Additionally, a number of animal studies indicate a role for GLO1 in CVD. In Apolipoprotein E-deficient (ApoE−/−) mice, an established mouse model of atherosclerosis, GLO1 inhibition by bromobenzyl-glutathione cyclopentyl diester increased vascular adhesion and augment atherogenesis [142]. In addition, GLO1 overexpression seemed to preserve cardiac function post-myocardial infarction by increasing vascularity [143]. Furthermore, GLO1 is downregulated in hypoxia linked to tissue ischemia [75,144]. Together these studies implicate an important role for GLO1 in CVD.

Several studies suggest a role for GLO1 in neurodegenerative disease, such as Alzheimer’s (AD) and Parkinson’s disease (PD) and it has been proposed that dicarbonyl stress may be a hallmark of AD [145]. GLO1 expression and activity declined in both advancing stage of AD and with increasing age in comparison to age-matched controls [117,118]. GLO1 levels decreased in old-aged human cortices compared to young brain. Elevated GLO1 levels were found in Alzheimer’s patients and GLO1 amount also decreased with age [119]. However, several animal models suggest that GLO1 expression is increased in neurodegenerative disease. GLO1 expression was increased in the brains of P301L mutant tau transgenic mice which develop neurofibrillary tangles, a histopathologic hallmark of AD and dementia [118]. Additionally, Ciavardelli et al. found increased GLO1 expression in the cerebellum of triple-transgenic AD mouse, which expresses mutant presinilin1 (M146V), amyloid precursor protein (swe), and tau (P301L) transgenes, and developed neuropathological progression of AD [146]. Experimental α-synuclein deficient PD mice had increased GLO1 expression in brain tissue compared to wild-type controls suggesting that α-synuclein may have a role in regulating MG-mediated dicarbonyl stress and thereby stress responsiveness increased in GLO1 expression [147].

Different studies have investigated a relationship between GLO1, brain function, behavior and psychosocial status. GLO1 has been associated with anxiety although the literature remains controversial. GLO1 duplications were associated with anxiety-like behavior in strains of laboratory mice [148]. Transgenic mice overexpressing *Glo1* displayed an anxiety phenotype. A decline in MG, a GABAA receptor agonist, in the brain by overexpression of GLO1 was proposed to explain the link to the anxiety state [105]. Kollmannsberger et al. observed that the insertion of *Glo1* duplication into a mouse model did not induce the anxiety phenotype in these mice [149], suggesting potential gene dosage thresholds. Previous literature pointed to both an association between increased GLO1 expression and anxiety; and the opposite, anxiety linked to decreased GLO1 expression [150,151]. GLO1 has also been linked to mood-affective disorders. An unusual clinical GLO1 deficiency, due to a rare frame shift mutation of GLO1, was associated with high risk of severe schizophrenia [26]. Another study showed that GLO1 expression of peripheral blood leukocytes declined in bipolar disorder patients [152]. A recent study showed that MG treatment decreased dopamine levels in the prefrontal cortex of mice along with a ~25% decrease in the GLO1 protein, resulting in memory deficits and depressive-like behavior [153]. Additionally, GLO1 protein and activity in brain tissues were reduced in the D-galactose-induced aging mouse model [154]. All of these studies suggest a relevant role of glyoxalase system in brain function.

GLO1 has been investigated and linked to studies of tumor growth and cancer therapy (reviewed in [155]). The human *GLO1* gene is a hotspot for copy number variation, as confirmed in a human population studies [156,157] and increasing *GLO1* expression associated with human tumors [158]. The glyoxalase system appears to have a dual role in cancer: a tumor-suppressor function in a healthy population and a mediator of multidrug resistance in established cancer. Briefly, tumor cells require a high rate of detoxification of MG to counteract the high glycolytic rate. GLO1 activity and expression is generally increased in many tumor cells. This reduces intracellular MG levels and avoids MG-induced apoptosis [20]. Indeed, experimental *GLO1* overexpression reduces intracellular MG levels, which decreases the activation of p38 MAPK-NFκB pathway, inhibiting the proapoptotic Bax and p53 proteins and, ultimately, enhancing the anti-apoptotic Bcl-2 protein expression [159,160]. Moreover, overexpression of GLO1 in human tumors is commonly linked to *Glo1* gene amplification [158]. *GLO1* overexpression can also be acquired by oncogene-linked malignant transformation [161] and chronic treatment with antitumor agents [162]. One of the first developed GLO1-inhibiting agents in tumor cells was S-p-bromobenzylglutathione cyclopentyl diester that leads to apoptosis in *GLO1*-overexpressing cancer cells [163,164].

### 3.2. Genetically Modified Models for the Study of Glyoxalase System Biology

A growing literature indicates that the accumulation of toxic AGEs impacts the cellular function in all tissues, playing a pathogenic role in the progression of molecular and cellular aging [109]. However, to date, suitable pharmacological tools to reduce the formation of AGEs or accelerate the AGEs degradation in a clinical context are lacking. For these reasons, there has been a significant effort to develop genetic models for the study of glyoxalase system function in vivo. Both cellular and animal models have been used to prove the causal involvement of MG and AGEs in multiple pathological disorders. In Table 1, relevant studies focused on the association between *Glo1* genetically modified cells and animal models are summarized (*Glo1* knockdown, KD; *Glo1* knockout, KO; *Glo1* overexpressed), accumulation of MG and toxic AGEs in tissues, modulation of lifespan in organisms and the prevention or development of age-related disease.

Regarding *Glo1* KD studies, there are several studies showing that the lack of GLO1 accelerates the accumulation of MG and MG-derived AGEs. The intracellular accumulation of MG by GLO1 KD was reported to disrupt collagen homoeostasis in L6 myoblasts [165]. Nigro et al. also found increased levels of MG and AGEs in GLO1 KD mouse aortic endothelial cells [166]. GLO1 KD in non-diabetic mice induced an alteration in glomerular proteins by MG, causing alterations in kidney morphology similar to diabetic nephropathy [127].

Recently, CRISPR-Cas technology allowed for the creation of viable *Glo1* KO organisms [167]. Curiously, some data in both KO cells and animal models showed no increased levels of MG in tissues, indicating that alternative routes, such as aldose reductases and DJ-1 might be able to increase catalytic efficiency and protect proteins from glycation [86,92,94,168]. Galligan et al. found that the lack of *GLO1* in HEK293 cells may induce deglycase activity of DJ-1, reducing MG-H1 deposition on chromatin [86] and Shumacher et al. observed increased aldose reductase activity in the liver and kidney of *Glo1* KO mice [94]. Increases in MG in tissues as well as MG-derived hydroimidazolone and AGE-derived proteins were observed in animals from which *Glo1* was KO [93,169]. Lipid accumulation and hyperglycemia later in life were also observed [169]. Similarly, overfeeding *Danio rerio Glo1* KO resulted in increased MG levels in tissue inducing hyperglycemia, insulin resistance and alteration in the retinal blood vessels [93].

Conversely, multiple studies using *Glo1* gene overexpression showed protective effects. Morcos et al. showed that the overexpression of *Glo1* enhanced lifespan in *Caenorhabditis elegans* and, reduced glyoxal and MG-derived hydroimidazolone, mitochondrial MG-derived protein modification as well as oxidative stress biomarkers [110]. In diabetes models, *Glo1* overexpression reduced glomerular protein modifications by MG, decreased oxidative stress markers, and prevented the development of diabetic kidney pathology [127]. Consistent with these observations, mice with *Glo1* overexpression had lower levels of MG in the brain [105]. Vulesevic et al. found a reduction of circulating biomarkers of inflammation, such as VCAM-1 (vascular cell adhesion molecule 1) and E-selectin (endothelial-leukocyte adhesion molecule 1) and diminished RAGE expression in diabetic *Glo1* overexpressing mice [170]. Other studies with rat models overexpressing GLO1 had similar results, showing a decreased concentration of glyoxal and MG-derived hydroimidazolone in tissues, reduction in AGE formation, and prevention of renal and endothelial dysfunction in response to induced diabetes [103,104,108]. Reduced damage was also observed in the diabetic retinal neuroglia and vascular cells with *Glo1* overexpression [108]. In cells overexpressing *Glo1*, MG levels decreased and delayed MG-induced protein modifications [171,172]. Finally, the upregulation of GLO1 and aldose reductase were protective against diabetic nephropathy alterations in diabetic patients [173] suggesting that boosting this system could be a possible therapeutic approach to delay the development of age-related diseases.

## 4. Nutritional Intervention to Enhance the Glyoxalase System and Decrease Accumulation of AGEs

There is an increasing interest in the discovery of small molecule regulators with the capacity to modulate glyoxalase system. He et al. recently summarized high-throughput microplate assays to identify novel regulators, chemical structures and the structure–activity relationship between GLO1 protein and modulators (reviewed in [174]). These studies are useful for drug design and the optimization of therapeutic doses for these compounds will require human clinical trials. However, dietary interventions based on supplements or changes in diet may be an alternative source of multiple glyoxalase activity enhancers to prevent or alleviate glycation-derived damage with age. In this section, we summarize the current status of knowledge about the anti-glycative activities of different nutritional compounds.

### 4.1. Isothiocyanates

Isothiocyanates are natural compounds formed by enzymatic conversion of glucosinolates, metabolites highly enriched in cruciferous vegetables. Sulforaphane and allyl isothiocyanate are two dietary isothiocyanates proven to enhance the activity of GLO1 in an NRF2-dependent manner and prevent glycative stress-induced DNA damage [65,71,175]. Proteomic analysis identified GLO1 as a protein differentially expressed in cells treated with sulforaphane [176]. Sulforaphane counteracts glycation-induced damage by lowering the phosphorylation of MAPK signaling pathways (ERK1/2, JNK, and p38) and, consequently, enhancing the glyoxalase system in primary neonatal rat cardiomyocytes and SH-SY5Y neuroblastoma cells [71,176]. Of note, the effect of sulforaphane could be cell-dependent, as the incubation of this isothiocyanates did not affect GLO1 activity in isolated peripheral blood mononuclear cells from healthy individuals [72].

### 4.2. Polyphenols

Polyphenols are natural compounds found in plant-based foods. They include flavonoids, phenolic acid, polyphenolic amides, and other polyphenols. Although a vast literature informs about the action of polyphenols on glyoxalase function, the role of polyphenols remains controversial. On one hand, studies report that some polyphenols can inhibit glyoxalase activity in vitro. Curcumin, a polyphenol commonly used as a food flavoring, as well as other flavonoids such as baicalein showed biological activity inhibiting GLO1 [177,178,179,180]. Oral administration of naringenin, a flavonoid present in citrus fruits, diminished the GLO1 enzymatic activity and increased AGEs intermediates in two mouse skin tumor model [181]. On the other hand, increasing literature reports that many of these micronutrients enhance GLO1 activity. Resveratrol, found in grapes, berries and peanuts, upregulates the glyoxalase system through the upregulation of ERK pathway and the nuclear translocation of NRF2. The depletion of NRF2 abrogates the resveratrol-induced upregulation of glyoxalase expression [182]. The effectiveness of resveratrol was demonstrated both in vivo upon oral treatment in streptozotocin-nicotinamide-induced diabetic rats and in vitro in several cell types [17,183,184]. A recent report adds new molecular clues and indicates that SIRT1 plays a role in the effective response of resveratrol against glycation-derived cytotoxicity [185]. Other polyphenols were also found to be bioactive in a glyoxalase-dependent manner. A polyphenol isolated from the edible seaweed *Ishige okamurae*, diphlorethohydroxycarmalol, inhibits glycative stress in vitro in a NRF2-dependent manner [186] and polyphenols from Chilean native berries also increased GLO1 activity and were cytoprotective in human gastric epithelial cells [187]. Recent reports have focused on the therapeutic potential of polyphenols in neurodegenerative disorders. Resveratrol, curcumin, capsaicin and epigallocatechin gallate induce neuroprotective effects through NRF2-induction in experimental models of Alzheimer’s and Parkinson’s disease (reviewed in [188,189]).

Flavonoids, the most common class of polyphenolic compounds in the human diet, are found in tea, citrus fruit, citrus fruit juices, berries, red wine, apples, and legumes. Flavonoids have attracted attention in the last decade, in part, due to their efficacy against glycative stress. Flavonoids are classified in different subgroups according to their chemical structure (anthocyanidins, anthoxanthins, flavanones, flavanonols and flavans) and members of almost every subclass have been reported to modulate GLO1.

Cyanidin belongs to the class of anthocyanidin and is found in many red berries and other fruits. Cyanidin exerts a protective effect against glycative stress by increasing the activity of GLO1 but with no changes in *Glo1* mRNA expression in rat pancreatic β-cells [190]. Anthocyanin have been reported to induce therapeutic activities in a wide range of disorders in the nervous systems, such as cerebral ischemia, Alzheimer’s disease and Parkinson’s disease [191]. Anthocyanins prevent neurotoxocity, but there is limited information about the impact of anthocyanins on glyoxalase system.

Within of the group of flavonoids, the class of anthoxanthins is likely the best characterized regarding antiglycation properties. However, the protective mechanism against glycative stress does not always correlate with modulation of glyoxalase system. Fisetin, a plant polyphenol found in many fruits and vegetables, promotes the expression and activity of GLO1, ameliorating major complications of diabetes in Akita mice, a model of type 1 diabetes [192]. Although reduced levels of glycated protein were found in different tissues of fisetin-treated animals, no significant change was detected in blood sugar. The impact of fisetin seems to be pleiotropic given that it also induced the expression of glutamate-cysteine ligase (GCL), the rate-limiting enzyme in GSH production, and reduced levels of RAGE receptor [192]. Quercetin also has anti-glycation properties by enhancing expression of GLO1 and GLO2 proteins, and increases cellular viability against MG-induced proteotoxicity in primary cultures of cerebellar neurons [193]. Other in vitro analysis showed that the protective effect of quercetin against high glucose-derived glycative stress is via NRF2/GLO1 pathway in neuroblastoma SH-SY5Y cells [175].

In vivo analyses corroborate in vitro experiments. Quercertin enhanced GLO1 in the brain of streptozotocin-induced diabetic rats [194] and lower plasma MG levels in a randomized, double-blind, placebo-controlled crossover trial [195]. However, neither quercetin nor epicatechin significantly changed the expression of GLO1 in peripheral blood mononuclear cells [195]. Compounds derived from the flavonol morin were recently shown to be protective in cultured mouse primary cerebellar neurons and *Caenorhabditis elegans* treated with MG. Increased NRF2, GLO1 and GLO2 expression, glyoxalase activity and GSH concentration were found in morin-treated models [196].

Flavanons comprise another type of flavonoid that modulates glyoxalase activity. Hesperetin, the main flavonoid in lemons and sweet oranges, activated the NRF2 pathway, enhanced GLO1 and ameliorated renal changes in streptozotocin-induced diabetic rats [197]. Of note, trans-resveratrol and hesperitin synergize to enhance expression and activity of GLO1 and inducing a decrease in glycated protein [198,199]. A trans-resveratrol and hesperetin formulation was shown to enhance angiogenesis and wound closure in diabetic mice [171]. Increased GLO1 activity in peripheral blood mononuclear cells was found in a randomized, placebo-controlled crossover clinical trial [198].

Genistein is an isoflavonol found in soy-based foods, coffee and other different plant-based foods. Dietary genistein enhanced the expression of GLO1 and GLO2 and significantly decreased MG and AGEs concentrations in two mouse models, fed with high-fat diet alone or in combination with MG [200].

In the subgroup of flavans, catechin, also enhanced the glyoxalase system activity and improved the detoxification of AGEs intermediates [193].

Xanthohumol, a prenylated flavonoid found in hops and beer, attenuated glycative stress in osteoblastic MC3T3-E1 cells by increasing NRF2 and GLO1 activity [201]. Different reports have shown neuroprotective properties of xanthohumol in murine neuroblastoma N2a cells stably expressing human Swedish mutant amyloid precursor protein and in ischemic stroke animal model [202]. However, it was not explored if the upregulation of GLO1 activity contributes to the neuroprotective effect. The xanthonoid mangiferin is another natural phenolic compound that upregulates NRF2 signaling, elevates glyoxalase activity, prevents the formation of AGEs and ameliorates diabetes-associated cognitive decline in rats [203,204,205].

### 4.3. Vitamins

Pyridoxamine, a form of vitamin B, has been shown to reduce glycative stress, by preventing the formation of MG-derived AGEs [206]. Enhanced erythrocyte GLO1 activity was found in streptozotocin-induced diabetic Sprague Dawley rats treated with pyridoxamine, which showed reduced formation of AGEs in plasma [206]. However, pyrodoxamine treatment failed to counteract AGE formation, did not lead to differences in GLO1 activity, or ameliorate disease progression in a model of experimental autoimmune encephalitis [207]. Changes in GLO1 expression were also not found in pyridoxamine-treated mice overexpressing inducible nitric oxide synthase in pancreatic β-cells [208]. The effect of pyridoxamine might be tissue dependent, as GLO1 expression was induced in visceral fat but not in perivascular fat tissues in Sprague Dawley rats fed a high fat diet [209].

Vitamin D was also reported to exert a protective role against glycative stress [210,211]. Vitamin D supplementation in type-2 diabetes participants showed a trend in increasing GLO1 expression, a transcriptional downregulation of RAGE receptor and decreased in AGE serum in a double-blind randomized placebo-controlled [211]. Nevertheless, the action of vitamins remains controversial because vitamin-treatment had no significant impact in other studies. For example, dietary vitamin E intake did not change mRNA *Glo1* levels and glutathione reductase 1 in the brain of inbred strains of mice [212]. In addition, vitamin D seemed to compensate for the loss of GLO1 in other diabetic contexts [213].

### 4.4. Other Dietary Compounds

Ursolic acid, found in the peels of fruits along with herbs and spices, showed an anti-glycative effect in the kidneys of diabetic mice. Enhanced renal Glo1 mRNA expression and glyoxalase activity were found in treated animals along with lower levels of plasma AGEs [214].

Some plant extracts used in traditional medicine in Asia have been evaluated. Monascin is a metabolite fermented by *Monascus purpureus*, a fungus used in the production of fermented food in Asia. Monascin is a natural PPARγ agonist that enhances GLO1 expression through activation of NRF2 and protects from glycative stress in rats orally administrated MG [215]. Extracts from *Psoralea corylifolia* seeds increased the expression of hepatic GLO1 in MG-treated mice [216]. Indole-4-carboxaldehyde isolated from the edible seaweed *Sargassum thunbergii* was also able to enhance mRNA and the expression of GLO1 in vitro [217].

Finally, the oral administration of glycine has been shown to promote the nuclear translocation of NRF2, thus protecting against glycative stress in different tissues. Glycine had a suppressive effect on glycative stress, manifested by increased activity and the expression of aortic GLO1 and reduced levels of AGEs [218]. Additionally, glycine enhanced the glyoxalase system function and protects against renal glycative stress in streptozotocin-induced diabetic rats [219].

## 5. Concluding Remarks and Pending Questions

AGEs are a common pathogenic factor for multiple age-related diseases, such as diverse as diabetes, AMD, CVD, neurocognitive and neurologic disorders, etc. The glyoxalase system is among the most robust protective capacities against formation of AGEs. Other mechanisms also seem to complement—or compensate for impaired—GLO1 activity. Although different protective mechanisms counteract the accumulation of these cytotoxic compounds, the activity of these cellular defenses decline with age and AGEs are gradually deposited, compromising aged tissues. Metabolic conditions such as diabetes and the consumption of Western, high glycemic diets exacerbate the AGE accumulation and represent a serious burden on public healthcare.

In order to diminish the detrimental effect of glycative stress on health, strategies extending the capacity of anti-AGE pathways with age should be salutary. The glyoxalase system is a primarily mechanism that limit the synthesis of AGEs and alterations of its activity is linked to multiple age-related diseases. Pharmacological interventions have not been successful in clinical setting so far. Dietary management or nutritional supplements may represent safe and low-cost alternatives to prolong the functionality of detoxifying routes such as glyoxalase system. Further research is required to define nutritional approaches to overcome the progression of age-related diseases associated with glycative stress.

## Figures and Tables

**Table 1:** Summary of relevant studies related to the glyoxalase 1 modulation in research on diabetes and aging.

Model	Organism/Cells	Phenotype	Reference
*Glo1* KD	L6 myoblasts	Intracellular accumulation of MG, GO and MG-related AGEs.	Stratmann et al.
	Mouse Aortic Endothelial Cells	Accumulation of MG, MG modified proteins and impaired angiogenesis.	Nigro et al.
	Non-Diabetic mice	Increases in AGEs, oxidative stress, and mimics diabetic nephropathy.	Giacco et al.
*Glo1* KO	CRISPR/Cas9 Mouse line	No increases in MG and MGH-1 under hyperglycemic condition. Aldose reductase activity increased in the liver and kidney of Glo1KO mice.	Schumacher et al.
	CRISPR/Cas9 HEK293 Cells	Glo1KO cells were more sensitive to MG toxicity than wild-type cells. DJ-1 protects histones from adduction by MG.	Galligan et al.
	CRISPR/Cas9 Mouse Schwann Cells	No accumulation of MG and MG modified proteins increased catalytic efficiency of aldose reductase.	Morgenstern et al
	Drosophila Melanogaster	Increases in MG and MGH-1 in tissues, decreases in insulin sensitivity, and lipid accumulation and hyperglycemia later in life.	Moraru et al.
	CRISPR/Cas9 Danio Renio	Under high nutrient intake, high MG levels, impaired glucose tolerance, and retinal blood vessel impairment.	Lodd et al.
*Glo1* OE	Caenorhabditis Elegans	Reduce levels of G-H1, CEL and MG-H1, and MG modifications of mitochondrial proteins; decrease in markers of oxidative damage; increase mean and maximum lifespan.	Morcos et al.
	Diabetic rat (STZ)	Less levels of MG, GO, AGEs and oxidative stress markers, and hyperglycemia reduction.	Brouwers et al.
	Diabetic rat (STZ)	Ameliorate CEL and MG-H1 accumulation in the diabetic retina and prevent alterations in retinal neuroglia and vascular cells.	Berner et al.
	Diabetic rat (STZ)	Decrease in MG levels, less AGE formation, and reduced renal and endothelial dysfunction in response to induced diabetes.	Brouwers et al.
	Bovine Retinal Endothelial Cells	Improvement of cell viability induced by MG and prevent mitochondrial protein glycated modification.	Qian et al.
	Diabetic mice (STZ)	Restored the circulating levels of inflammatory markers (E-selectin, V-CAM), reduced RAGE expression and MG-induced endothelial cell loss.	Vulesevic et al.
	Mouse Bone Marrow-derived Progenitor Cells (db/db)	Prevent MG-impairment of IRE1α expression and activity in diabetic mice.	Hainan et al.

## Data Availability

The data presented in this study are available on request from the corresponding author.

## Supplementary Materials

The following are available online at https://www.mdpi.com/article/10.3390/cells10081852/s1, Figure S1: Differential expression of GLO1 in mouse tissues.

## References

[1] Prasad C., Imrhan V., Marotta F., Juma S., Vijayagopal P. (2014). Lifestyle and Advanced Glycation End Products (AGEs) Burden: Its Relevance to Healthy Aging. Aging Dis..

[2] Rowan S., Bejarano E., Taylor A. (2018). Mechanistic targeting of advanced glycation end-products in age-related diseases. Biochim. Biophys. Acta. Mol. Basis Dis..

[3] Kandarakis S.A., Piperi C., Topouzis F., Papavassiliou A.G. (2014). Emerging role of advanced glycation-end products (AGEs) in the pathobiology of eye diseases. Prog. Retin. Eye Res..

[4] Semba R.D., Nicklett E.J., Ferrucci L. (2010). Does accumulation of advanced glycation end products contribute to the aging phenotype?. J. Gerontol. Ser. A Biol. Sci. Med. Sci..

[5] Taylor A. (2012). Mechanistically linking age-related diseases and dietary carbohydrate via autophagy and the ubiquitin proteolytic systems. Autophagy.

[6] Jahngen J.H., Lipman R.D., Eisenhauer D.A., Jahngen E.G., Taylor A. (1990). Aging and cellular maturation cause changes in ubiquitin-eye lens protein conjugates. Arch. Biochem. Biophys..

[7] Jahngen-Hodge J., Cyr D., Laxman E., Taylor A. (1992). Ubiquitin and ubiquitin conjugates in human lens. Exp. Eye Res..

[8] Thornalley P.J., Yurek-George A., Argirov O.K. (2000). Kinetics and mechanism of the reaction of aminoguanidine with the alpha-oxoaldehydes glyoxal, methylglyoxal, and 3-deoxyglucosone under physiological conditions. Biochem. Pharmacol..

[9] Uchiki T., Weikel K.A., Jiao W., Shang F., Caceres A., Pawlak D., Handa J.T., Brownlee M., Nagaraj R., Taylor A. (2012). Glycation-altered proteolysis as a pathobiologic mechanism that links dietary glycemic index, aging, and age-related disease (in nondiabetics). Aging Cell.

[10] Vlassara H., Striker G.E. (2011). AGE restriction in diabetes mellitus: A paradigm shift. Nat. Rev. Endocrinol..

[11] Cai W., Uribarri J., Zhu L., Chen X., Swamy S., Zhao Z., Grosjean F., Simonaro C., Kuchel G.A., Schnaider-Beeri M. (2014). Oral glycotoxins are a modifiable cause of dementia and the metabolic syndrome in mice and humans. Proc. Natl. Acad. Sci. USA.

[12] Uribarri J., Cai W., Ramdas M., Goodman S., Pyzik R., Chen X., Zhu L., Striker G.E., Vlassara H. (2011). Restriction of advanced glycation end products improves insulin resistance in human type 2 diabetes: Potential role of AGER1 and SIRT1. Diabetes Care.

[13] Beeri M.S., Moshier E., Schmeidler J., Godbold J., Uribarri J., Reddy S., Sano M., Grossman H.T., Cai W., Vlassara H. (2011). Serum concentration of an inflammatory glycotoxin, methylglyoxal, is associated with increased cognitive decline in elderly individuals. Mech. Ageing Dev..

[14] Sruthi C.R., Raghu K.G. (2021). Advanced glycation end products and their adverse effects: The role of autophagy. J. Biochem. Mol. Toxicol..

[15] Bierhaus A., Fleming T., Stoyanov S., Leffler A., Babes A., Neacsu C., Sauer S.K., Eberhardt M., Schnölzer M., Lasitschka F. (2012). Methylglyoxal modification of Nav1.8 facilitates nociceptive neuron firing and causes hyperalgesia in diabetic neuropathy. Nat. Med..

[16] Barati M.T., Merchant M.L., Kain A.B., Jevans A.W., McLeish K.R., Klein J.B. (2007). Proteomic analysis defines altered cellular redox pathways and advanced glycation end-product metabolism in glomeruli of db/db diabetic mice. Am. J. Physiol. Ren. Physiol..

[17] Palsamy P., Subramanian S. (2011). Resveratrol protects diabetic kidney by attenuating hyperglycemia-mediated oxidative stress and renal inflammatory cytokines via Nrf2-Keap1 signaling. Biochim. Biophys. Acta.

[18] Miller A.G., Tan G., Binger K.J., Pickering R.J., Thomas M.C., Nagaraj R.H., Cooper M.E., Wilkinson-Berka J.L. (2010). Candesartan attenuates diabetic retinal vascular pathology by restoring glyoxalase-I function. Diabetes.

[19] Karachalias N., Babaei-Jadidi R., Rabbani N., Thornalley P.J. (2010). Increased protein damage in renal glomeruli, retina, nerve, plasma and urine and its prevention by thiamine and benfotiamine therapy in a rat model of diabetes. Diabetologia.

[20] Maessen D.E., Stehouwer C.D., Schalkwijk C.G. (2015). The role of methylglyoxal and the glyoxalase system in diabetes and other age-related diseases. Clin. Sci..

[21] Schalkwijk C.G., Stehouwer C.D.A. (2020). Methylglyoxal, a Highly Reactive Dicarbonyl Compound, in Diabetes, Its Vascular Complications, and Other Age-Related Diseases. Physiol. Rev..

[22] Hanssen N.M., Wouters K., Huijberts M.S., Gijbels M.J., Sluimer J.C., Scheijen J.L., Heeneman S., Biessen E.A., Daemen M.J., Brownlee M. (2014). Higher levels of advanced glycation endproducts in human carotid atherosclerotic plaques are associated with a rupture-prone phenotype. Eur. Heart J..

[23] Ahmed N., Thornalley P.J. (2005). Peptide mapping of human serum albumin modified minimally by methylglyoxal in vitro and in vivo. Ann. N. Y. Acad. Sci..

[24] Wang X., Desai K., Clausen J.T., Wu L. (2004). Increased methylglyoxal and advanced glycation end products in kidney from spontaneously hypertensive rats. Kidney Int..

[25] Wang X., Desai K., Chang T., Wu L. (2005). Vascular methylglyoxal metabolism and the development of hypertension. J. Hypertens..

[26] Arai M., Yuzawa H., Nohara I., Ohnishi T., Obata N., Iwayama Y., Haga S., Toyota T., Ujike H., Arai M. (2010). Enhanced carbonyl stress in a subpopulation of schizophrenia. Arch. Gen. Psychiatry.

[27] Ahmed N., Ahmed U., Thornalley P.J., Hager K., Fleischer G., Münch G. (2005). Protein glycation, oxidation and nitration adduct residues and free adducts of cerebrospinal fluid in Alzheimer’s disease and link to cognitive impairment. J. Neurochem..

[28] Vicente Miranda H., Szego É.M., Oliveira L.M.A., Breda C., Darendelioglu E., de Oliveira R.M., Ferreira D.G., Gomes M.A., Rott R., Oliveira M. (2017). Glycation potentiates α-synuclein-associated neurodegeneration in synucleinopathies. Brain A J. Neurol..

[29] Bejarano E., Taylor A. (2019). Too sweet: Problems of protein glycation in the eye. Exp. Eye Res..

[30] Aragonès G., Rowan S., Francisco S.G., Yang W., Weinberg J., Taylor A., Bejarano E. (2020). Glyoxalase System as a Therapeutic Target against Diabetic Retinopathy. Antioxidants.

[31] Tessier F., Obrenovich M., Monnier V.M. (1999). Structure and mechanism of formation of human lens fluorophore LM-1. Relationship to vesperlysine A and the advanced Maillard reaction in aging, diabetes, and cataractogenesis. J. Biol. Chem..

[32] Hammes H.P., Hoerauf H., Alt A., Schleicher E., Clausen J.T., Bretzel R.G., Laqua H. (1999). N(epsilon)(carboxymethyl)lysin and the AGE receptor RAGE colocalize in age-related macular degeneration. Investig. Ophthalmol. Vis. Sci..

[33] Handa J.T., Verzijl N., Matsunaga H., Aotaki-Keen A., Lutty G.A., te Koppele J.M., Miyata T., Hjelmeland L.M. (1999). Increase in the advanced glycation end product pentosidine in Bruch’s membrane with age. Investig. Ophthalmol. Vis. Sci..

[34] Weikel K.A., Fitzgerald P., Shang F., Caceres M.A., Bian Q., Handa J.T., Stitt A.W., Taylor A. (2012). Natural history of age-related retinal lesions that precede AMD in mice fed high or low glycemic index diets. Investig. Ophthalmol. Vis. Sci..

[35] Rowan S., Jiang S., Korem T., Szymanski J., Chang M.L., Szelog J., Cassalman C., Dasuri K., McGuire C., Nagai R. (2017). Involvement of a gut-retina axis in protection against dietary glycemia-induced age-related macular degeneration. Proc. Natl. Acad. Sci. USA.

[36] Rowan S., Jiang S., Chang M.L., Volkin J., Cassalman C., Smith K.M., Streeter M.D., Spiegel D.A., Moreira-Neto C., Rabbani N. (2020). A low glycemic diet protects disease-prone Nrf2-deficient mice against age-related macular degeneration. Free. Radic. Biol. Med..

[37] Stitt A.W. (2010). AGEs and diabetic retinopathy. Investig. Ophthalmol. Vis. Sci..

[38] Engelen L., Stehouwer C.D., Schalkwijk C.G. (2013). Current therapeutic interventions in the glycation pathway: Evidence from clinical studies. Diabetes Obes. Metab..

[39] Brings S., Fleming T., Freichel M., Muckenthaler M.U., Herzig S., Nawroth P.P. (2017). Dicarbonyls and Advanced Glycation End-Products in the Development of Diabetic Complications and Targets for Intervention. Int. J. Mol. Sci..

[40] Takahashi A., Takabatake Y., Kimura T., Maejima I., Namba T., Yamamoto T., Matsuda J., Minami S., Kaimori J.Y., Matsui I. (2017). Autophagy Inhibits the Accumulation of Advanced Glycation End Products by Promoting Lysosomal Biogenesis and Function in the Kidney Proximal Tubules. Diabetes.

[41] Aragonès G., Dasuri K., Olukorede O., Francisco S.G., Renneburg C., Kumsta C., Hansen M., Kageyama S., Komatsu M., Rowan S. (2020). Autophagic receptor p62 protects against glycation-derived toxicity and enhances viability. Aging Cell.

[42] Gavilán E., Pintado C., Gavilan M.P., Daza P., Sánchez-Aguayo I., Castaño A., Ruano D. (2015). Age-related dysfunctions of the autophagy lysosomal pathway in hippocampal pyramidal neurons under proteasome stress. Neurobiol. Aging.

[43] Quinet G., Gonzalez-Santamarta M., Louche C., Rodriguez M.S. (2020). Mechanisms Regulating the UPS-ALS Crosstalk: The Role of Proteaphagy. Molecules.

[44] Shin W.H., Park J.H., Chung K.C. (2020). The central regulator p62 between ubiquitin proteasome system and autophagy and its role in the mitophagy and Parkinson’s disease. BMB Rep..

[45] Kocaturk N.M., Gozuacik D. (2018). Crosstalk Between Mammalian Autophagy and the Ubiquitin-Proteasome System. Front. Cell Dev. Biol..

[46] Ji C.H., Kwon Y.T. (2017). Crosstalk and Interplay between the Ubiquitin-Proteasome System and Autophagy. Mol. Cells.

[47] Kwon Y.T., Ciechanover A. (2017). The Ubiquitin Code in the Ubiquitin-Proteasome System and Autophagy. Trends Biochem. Sci..

[48] Makrides S.C. (1983). Protein synthesis and degradation during aging and senescence. Biol. Rev. Camb. Philos. Soc..

[49] Martinez-Vicente M., Sovak G., Cuervo A.M. (2005). Protein degradation and aging. Exp. Gerontol..

[50] Keller J.N., Dimayuga E., Chen Q., Thorpe J., Gee J., Ding Q. (2004). Autophagy, proteasomes, lipofuscin, and oxidative stress in the aging brain. Int. J. Biochem. Cell Biol..

[51] Cuervo A.M., Macian F. (2014). Autophagy and the immune function in aging. Curr. Opin. Immunol..

[52] Bejarano E., Murray J.W., Wang X., Pampliega O., Yin D., Patel B., Yuste A., Wolkoff A.W., Cuervo A.M. (2018). Defective recruitment of motor proteins to autophagic compartments contributes to autophagic failure in aging. Aging Cell.

[53] Carrard G., Bulteau A.L., Petropoulos I., Friguet B. (2002). Impairment of proteasome structure and function in aging. Int. J. Biochem. Cell Biol..

[54] Ferrington D.A., Husom A.D., Thompson L.V. (2005). Altered proteasome structure, function, and oxidation in aged muscle. FASEB J..

[55] Cuervo A.M. (2008). Autophagy and aging: Keeping that old broom working. Trends Genet. TIG.

[56] Shang F., Taylor A. (2012). Roles for the ubiquitin-proteasome pathway in protein quality control and signaling in the retina: Implications in the pathogenesis of age-related macular degeneration. Mol. Asp. Med..

[57] Moaddel R., Ubaida-Mohien C., Tanaka T., Lyashkov A., Basisty N., Schilling B., Semba R.D., Franceschi C., Gorospe M., Ferrucci L. (2021). Proteomics in aging research: A roadmap to clinical, translational research. Aging Cell.

[58] Thornalley P.J. (1990). The glyoxalase system: New developments towards functional characterization of a metabolic pathway fundamental to biological life. Biochem. J..

[59] Abordo E.A., Minhas H.S., Thornalley P.J. (1999). Accumulation of alpha-oxoaldehydes during oxidative stress: A role in cytotoxicity. Biochem. Pharmacol..

[60] Vander Jagt D.L., Han L.P., Lehman C.H. (1972). Kinetic evaluation of substrate specificity in the glyoxalase-I-catalyzed disproportionation of -ketoaldehydes. Biochemistry.

[61] Shinohara M., Thornalley P.J., Giardino I., Beisswenger P., Thorpe S.R., Onorato J., Brownlee M. (1998). Overexpression of glyoxalase-I in bovine endothelial cells inhibits intracellular advanced glycation endproduct formation and prevents hyperglycemia-induced increases in macromolecular endocytosis. J. Clin. Investig..

[62] Ranganathan S., Ciaccio P.J., Walsh E.S., Tew K.D. (1999). Genomic sequence of human glyoxalase-I: Analysis of promoter activity and its regulation. Gene.

[63] Conboy C.M., Spyrou C., Thorne N.P., Wade E.J., Barbosa-Morais N.L., Wilson M.D., Bhattacharjee A., Young R.A., Tavaré S., Lees J.A. (2007). Cell cycle genes are the evolutionarily conserved targets of the E2F4 transcription factor. PLoS ONE.

[64] Orso F., Corà D., Ubezio B., Provero P., Caselle M., Taverna D. (2010). Identification of functional TFAP2A and SP1 binding sites in new TFAP2A-modulated genes. BMC Genom..

[65] Xue M., Rabbani N., Momiji H., Imbasi P., Anwar M.M., Kitteringham N., Park B.K., Souma T., Moriguchi T., Yamamoto M. (2012). Transcriptional control of glyoxalase 1 by Nrf2 provides a stress-responsive defence against dicarbonyl glycation. Biochem. J..

[66] Itoh K., Ishii T., Wakabayashi N., Yamamoto M. (1999). Regulatory mechanisms of cellular response to oxidative stress. Free. Radic. Res..

[67] Cullinan S.B., Gordan J.D., Jin J., Harper J.W., Diehl J.A. (2004). The Keap1-BTB protein is an adaptor that bridges Nrf2 to a Cul3-based E3 ligase: Oxidative stress sensing by a Cul3-Keap1 ligase. Mol. Cell. Biol..

[68] Nguyen T., Sherratt P.J., Nioi P., Yang C.S., Pickett C.B. (2005). Nrf2 controls constitutive and inducible expression of ARE-driven genes through a dynamic pathway involving nucleocytoplasmic shuttling by Keap1. J. Biol. Chem..

[69] Bollong M.J., Lee G., Coukos J.S., Yun H., Zambaldo C., Chang J.W., Chin E.N., Ahmad I., Chatterjee A.K., Lairson L.L. (2018). A metabolite-derived protein modification integrates glycolysis with KEAP1-NRF2 signalling. Nature.

[70] James D., Devaraj S., Bellur P., Lakkanna S., Vicini J., Boddupalli S. (2012). Novel concepts of broccoli sulforaphanes and disease: Induction of phase II antioxidant and detoxification enzymes by enhanced-glucoraphanin broccoli. Nutr. Rev..

[71] Angeloni C., Malaguti M., Rizzo B., Barbalace M.C., Fabbri D., Hrelia S. (2015). Neuroprotective effect of sulforaphane against methylglyoxal cytotoxicity. Chem. Res. Toxicol..

[72] Alfarano M., Pastore D., Fogliano V., Schalkwijk C.G., Oliviero T. (2018). The Effect of Sulforaphane on Glyoxalase I Expression and Activity in Peripheral Blood Mononuclear Cells. Nutrients.

[73] Pereira A., Fernandes R., Crisóstomo J., Seiça R.M., Sena C.M. (2017). The Sulforaphane and pyridoxamine supplementation normalize endothelial dysfunction associated with type 2 diabetes. Sci. Rep..

[74] Liu G.H., Qu J., Shen X. (2008). NF-kappaB/p65 antagonizes Nrf2-ARE pathway by depriving CBP from Nrf2 and facilitating recruitment of HDAC3 to MafK. Biochim. Biophys. Acta.

[75] Zhang H., Li H., Xi H.S., Li S. (2012). HIF1α is required for survival maintenance of chronic myeloid leukemia stem cells. Blood.

[76] Rauh D., Fischer F., Gertz M., Lakshminarasimhan M., Bergbrede T., Aladini F., Kambach C., Becker C.F., Zerweck J., Schutkowski M. (2013). An acetylome peptide microarray reveals specificities and deacetylation substrates for all human sirtuin isoforms. Nat. Commun..

[77] Lundby A., Lage K., Weinert B.T., Bekker-Jensen D.B., Secher A., Skovgaard T., Kelstrup C.D., Dmytriyev A., Choudhary C., Lundby C. (2012). Proteomic analysis of lysine acetylation sites in rat tissues reveals organ specificity and subcellular patterns. Cell Rep..

[78] Reiniger N., Lau K., McCalla D., Eby B., Cheng B., Lu Y., Qu W., Quadri N., Ananthakrishnan R., Furmansky M. (2010). Deletion of the receptor for advanced glycation end products reduces glomerulosclerosis and preserves renal function in the diabetic OVE26 mouse. Diabetes.

[79] Morgenstern J., Katz S., Krebs-Haupenthal J., Chen J., Saadatmand A., Cortizo F.G., Moraru A., Zemva J., Campos M.C., Teleman A. (2020). Phosphorylation of T107 by CamKIIδ Regulates the Detoxification Efficiency and Proteomic Integrity of Glyoxalase 1. Cell Rep..

[80] Kold-Christensen R., Johannsen M. (2020). Methylglyoxal Metabolism and Aging-Related Disease: Moving from Correlation toward Causation. Trends Endocrinol. Metab. TEM.

[81] Bonifati V., Rizzu P., van Baren M.J., Schaap O., Breedveld G.J., Krieger E., Dekker M.C., Squitieri F., Ibanez P., Joosse M. (2003). Mutations in the DJ-1 gene associated with autosomal recessive early-onset parkinsonism. Science.

[82] Lev N., Roncevic D., Ickowicz D., Melamed E., Offen D. (2006). Role of DJ-1 in Parkinson’s disease. J. Mol. Neurosci. MN.

[83] Lee J.Y., Song J., Kwon K., Jang S., Kim C., Baek K., Kim J., Park C. (2012). Human DJ-1 and its homologs are novel glyoxalases. Hum. Mol. Genet..

[84] Richarme G., Mihoub M., Dairou J., Bui L.C., Leger T., Lamouri A. (2015). Parkinsonism-associated protein DJ-1/Park7 is a major protein deglycase that repairs methylglyoxal- and glyoxal-glycated cysteine, arginine, and lysine residues. J. Biol. Chem..

[85] Richarme G., Liu C., Mihoub M., Abdallah J., Leger T., Joly N., Liebart J.C., Jurkunas U.V., Nadal M., Bouloc P. (2017). Guanine glycation repair by DJ-1/Park7 and its bacterial homologs. Science.

[86] Galligan J.J., Wepy J.A., Streeter M.D., Kingsley P.J., Mitchener M.M., Wauchope O.R., Beavers W.N., Rose K.L., Wang T., Spiegel D.A. (2018). Methylglyoxal-derived posttranslational arginine modifications are abundant histone marks. Proc. Natl. Acad. Sci. USA.

[87] Zheng Q., Omans N.D., Leicher R., Osunsade A., Agustinus A.S., Finkin-Groner E., D’Ambrosio H., Liu B., Chandarlapaty S., Liu S. (2019). Reversible histone glycation is associated with disease-related changes in chromatin architecture. Nat. Commun..

[88] Pfaff D.H., Fleming T., Nawroth P., Teleman A.A. (2017). Evidence Against a Role for the Parkinsonism-associated Protein DJ-1 in Methylglyoxal Detoxification. J. Biol. Chem..

[89] Li D., Ferrari M., Ellis E.M. (2012). Human aldo-keto reductase AKR7A2 protects against the cytotoxicity and mutagenicity of reactive aldehydes and lowers intracellular reactive oxygen species in hamster V79-4 cells. Chem.-Biol. Interact..

[90] Li D., Ellis E.M. (2014). Aldo-keto reductase 7A5 (AKR7A5) attenuates oxidative stress and reactive aldehyde toxicity in V79-4 cells. Toxicol. Vitr. Int. J. Publ. Assoc. BIBRA.

[91] Baba S.P., Barski O.A., Ahmed Y., O’Toole T.E., Conklin D.J., Bhatnagar A., Srivastava S. (2009). Reductive metabolism of AGE precursors: A metabolic route for preventing AGE accumulation in cardiovascular tissue. Diabetes.

[92] Morgenstern J., Fleming T., Schumacher D., Eckstein V., Freichel M., Herzig S., Nawroth P. (2017). Loss of Glyoxalase 1 Induces Compensatory Mechanism to Achieve Dicarbonyl Detoxification in Mammalian Schwann Cells. J. Biol. Chem..

[93] Lodd E., Wiggenhauser L.M., Morgenstern J., Fleming T.H., Poschet G., Büttner M., Tabler C.T., Wohlfart D.P., Nawroth P.P., Kroll J. (2019). The combination of loss of glyoxalase1 and obesity results in hyperglycemia. JCI Insight.

[94] Schumacher D., Morgenstern J., Oguchi Y., Volk N., Kopf S., Groener J.B., Nawroth P.P., Fleming T., Freichel M. (2018). Compensatory mechanisms for methylglyoxal detoxification in experimental & clinical diabetes. Mol. Metab..

[95] Kalapos M.P. (2003). On the mammalian acetone metabolism: From chemistry to clinical implications. Biochim. Biophys. Acta.

[96] Salomón T., Sibbersen C., Hansen J., Britz D., Svart M.V., Voss T.S., Møller N., Gregersen N., Jørgensen K.A., Palmfeldt J. (2017). Ketone Body Acetoacetate Buffers Methylglyoxal via a Non-enzymatic Conversion during Diabetic and Dietary Ketosis. Cell Chem. Biol..

[97] Spencer P.S., Chen X. (2021). The Role of Protein Adduction in Toxic Neuropathies of Exogenous and Endogenous Origin. Toxics.

[98] Boekelheide K., Fleming S.L., Allio T., Embree-Ku M.E., Hall S.J., Johnson K.J., Kwon E.J., Patel S.R., Rasoulpour R.J., Schoenfeld H.A. (2003). 2,5-hexanedione-induced testicular injury. Annu. Rev. Pharmacol. Toxicol..

[99] Jain D., Jain R., Eberhard D., Eglinger J., Bugliani M., Piemonti L., Marchetti P., Lammert E. (2012). Age- and diet-dependent requirement of DJ-1 for glucose homeostasis in mice with implications for human type 2 diabetes. J. Mol. Cell Biol..

[100] Hong Z., Shi M., Chung K.A., Quinn J.F., Peskind E.R., Galasko D., Jankovic J., Zabetian C.P., Leverenz J.B., Baird G. (2010). DJ-1 and alpha-synuclein in human cerebrospinal fluid as biomarkers of Parkinson’s disease. Brain A J. Neurol..

[101] Gu X., Neric N.J., Crabb J.S., Crabb J.W., Bhattacharya S.K., Rayborn M.E., Hollyfield J.G., Bonilha V.L. (2012). Age-related changes in the retinal pigment epithelium (RPE). PLoS ONE.

[102] Arai M., Nihonmatsu-Kikuchi N., Itokawa M., Rabbani N., Thornalley P.J. (2014). Measurement of glyoxalase activities. Biochem. Soc. Trans..

[103] Brouwers O., Niessen P.M., Ferreira I., Miyata T., Scheffer P.G., Teerlink T., Schrauwen P., Brownlee M., Stehouwer C.D., Schalkwijk C.G. (2011). Overexpression of glyoxalase-I reduces hyperglycemia-induced levels of advanced glycation end products and oxidative stress in diabetic rats. J. Biol. Chem..

[104] Brouwers O., Niessen P.M., Miyata T., Østergaard J.A., Flyvbjerg A., Peutz-Kootstra C.J., Sieber J., Mundel P.H., Brownlee M., Janssen B.J. (2014). Glyoxalase-1 overexpression reduces endothelial dysfunction and attenuates early renal impairment in a rat model of diabetes. Diabetologia.

[105] Distler M.G., Plant L.D., Sokoloff G., Hawk A.J., Aneas I., Wuenschell G.E., Termini J., Meredith S.C., Nobrega M.A., Palmer A.A. (2012). Glyoxalase 1 increases anxiety by reducing GABAA receptor agonist methylglyoxal. J. Clin. Investig..

[106] Chen S.M., Lin C.E., Chen H.H., Cheng Y.F., Cheng H.W., Imai K. (2020). Effect of prednisolone on glyoxalase 1 in an inbred mouse model of aristolochic acid nephropathy using a proteomics method with fluorogenic derivatization-liquid chromatography-tandem mass spectrometry. PLoS ONE.

[107] Miller A.G., Smith D.G., Bhat M., Nagaraj R.H. (2006). Glyoxalase I is critical for human retinal capillary pericyte survival under hyperglycemic conditions. J. Biol. Chem..

[108] Berner A.K., Brouwers O., Pringle R., Klaassen I., Colhoun L., McVicar C., Brockbank S., Curry J.W., Miyata T., Brownlee M. (2012). Protection against methylglyoxal-derived AGEs by regulation of glyoxalase 1 prevents retinal neuroglial and vasodegenerative pathology. Diabetologia.

[109] Rabbani N., Xue M., Thornalley P.J. (2016). Dicarbonyls and glyoxalase in disease mechanisms and clinical therapeutics. Glycoconj. J..

[110] Morcos M., Du X., Pfisterer F., Hutter H., Sayed A.A., Thornalley P., Ahmed N., Baynes J., Thorpe S., Kukudov G. (2008). Glyoxalase-1 prevents mitochondrial protein modification and enhances lifespan in Caenorhabditis elegans. Aging Cell.

[111] Sharma-Luthra R., Kale R.K. (1994). Age related changes in the activity of the glyoxalase system. Mech. Ageing Dev..

[112] Amicarelli F., Di Ilio C., Masciocco L., Bonfigli A., Zarivi O., D’Andrea M.R., Di Giulio C., Miranda M. (1997). Aging and detoxifying enzymes responses to hypoxic or hyperoxic treatment. Mech. Ageing Dev..

[113] Kirk J.E. (1960). The glyoxalase I activity of arterial tissue in individuals of various ages. J. Gerontol..

[114] Haik G.M., Lo T.W., Thornalley P.J. (1994). Methylglyoxal concentration and glyoxalase activities in the human lens. Exp. Eye Res..

[115] Mailankot M., Padmanabha S., Pasupuleti N., Major D., Howell S., Nagaraj R.H. (2009). Glyoxalase I activity and immunoreactivity in the aging human lens. Biogerontology.

[116] McLellan A.C., Thornalley P.J. (1989). Glyoxalase activity in human red blood cells fractioned by age. Mech. Ageing Dev..

[117] Kuhla B., Boeck K., Lüth H.J., Schmidt A., Weigle B., Schmitz M., Ogunlade V., Münch G., Arendt T. (2006). Age-dependent changes of glyoxalase I expression in human brain. Neurobiol. Aging.

[118] Chen F., Wollmer M.A., Hoerndli F., Münch G., Kuhla B., Rogaev E.I., Tsolaki M., Papassotiropoulos A., Götz J. (2004). Role for glyoxalase I in Alzheimer’s disease. Proc. Natl. Acad. Sci. USA.

[119] Kuhla B., Boeck K., Schmidt A., Ogunlade V., Arendt T., Münch G., Lüth H.J. (2007). Age- and stage-dependent glyoxalase I expression and its activity in normal and Alzheimer’s disease brains. Neurobiol. Aging.

[120] Rankinen T., Zuberi A., Chagnon Y.C., Weisnagel S.J., Argyropoulos G., Walts B., Pérusse L., Bouchard C. (2006). The human obesity gene map: The 2005 update. Obesity.

[121] Rabbani N., Thornalley P.J. (2019). Glyoxalase 1 Modulation in Obesity and Diabetes. Antioxid. Redox Signal..

[122] Wuschke S., Dahm S., Schmidt C., Joost H.G., Al-Hasani H. (2007). A meta-analysis of quantitative trait loci associated with body weight and adiposity in mice. Int. J. Obes..

[123] Wilson A.F., Elston R.C., Tran L.D., Siervogel R.M. (1991). Use of the robust sib-pair method to screen for single-locus, multiple-locus, and pleiotropic effects: Application to traits related to hypertension. Am. J. Hum. Genet..

[124] Sanchez J.C., Converset V., Nolan A., Schmid G., Wang S., Heller M., Sennitt M.V., Hochstrasser D.F., Cawthorne M.A. (2002). Effect of rosiglitazone on the differential expression of diabetes-associated proteins in pancreatic islets of C57Bl/6 lep/lep mice. Mol. Cell. Proteom. MCP.

[125] Wortmann M., Peters A., Hakimi M., Bockler D., Dihlmann S. (2014). Glyoxalase I (Glo1) and its metabolites in vascular disease. Biochem. Soc. Trans..

[126] Maessen D., Brouwers O., Miyata T., Stehouwer C., Schalkwijk C. (2014). Glyoxalase-1 overexpression reduces body weight and adipokine expression, and improves insulin sensitivity in high-fat diet-induced obese mice. Diabetologia.

[127] Giacco F., Du X., D’Agati V.D., Milne R., Sui G., Geoffrion M., Brownlee M. (2014). Knockdown of glyoxalase 1 mimics diabetic nephropathy in nondiabetic mice. Diabetes.

[128] Yao D., Brownlee M. (2010). Hyperglycemia-induced reactive oxygen species increase expression of the receptor for advanced glycation end products (RAGE) and RAGE ligands. Diabetes.

[129] Dobler D., Ahmed N., Song L., Eboigbodin K.E., Thornalley P.J. (2006). Increased dicarbonyl metabolism in endothelial cells in hyperglycemia induces anoikis and impairs angiogenesis by RGD and GFOGER motif modification. Diabetes.

[130] Phillips S.A., Mirrlees D., Thornalley P.J. (1993). Modification of the glyoxalase system in streptozotocin-induced diabetic rats. Effect of the aldose reductase inhibitor Statil. Biochem. Pharmacol..

[131] Atkins T.W., Thornally P.J. (1989). Erythrocyte glyoxalase activity in genetically obese (ob/ob) and streptozotocin diabetic mice. Diabetes Res..

[132] Chiu C.J., Liu S., Willett W.C., Wolever T.M., Brand-Miller J.C., Barclay A.W., Taylor A. (2011). Informing food choices and health outcomes by use of the dietary glycemic index. Nutr. Rev..

[133] Francisco S.G., Smith K.M., Aragonès G., Whitcomb E.A., Weinberg J., Wang X., Bejarano E., Taylor A., Rowan S. (2020). Dietary Patterns, Carbohydrates, and Age-Related Eye Diseases. Nutrients.

[134] Chiu C.J., Taylor A. (2011). Dietary hyperglycemia, glycemic index and metabolic retinal diseases. Prog. Retin. Eye Res..

[135] Rasul A., Rashid A., Waheed P., Khan S.A. (2018). Expression analysis of glyoxalase I gene among patients of diabetic retinopathy. Pak. J. Med Sci..

[136] Zaidi A., Waheed P., Rashid A., Khan S.A. (2018). Gene Expression of Glyoxalase II in Diabetic Retinopathy. J. Coll. Physicians Surg. Pak. JCPSP.

[137] Wu J.C., Li X.H., Peng Y.D., Wang J.B., Tang J.F., Wang Y.F. (2011). Association of two glyoxalase I gene polymorphisms with nephropathy and retinopathy in Type 2 diabetes. J. Endocrinol. Investig..

[138] Sachdeva R., Schlotterer A., Schumacher D., Matka C., Mathar I., Dietrich N., Medert R., Kriebs U., Lin J., Nawroth P. (2018). TRPC proteins contribute to development of diabetic retinopathy and regulate glyoxalase 1 activity and methylglyoxal accumulation. Mol. Metab..

[139] Mäkinen V.P., Civelek M., Meng Q., Zhang B., Zhu J., Levian C., Huan T., Segrè A.V., Ghosh S., Vivar J. (2014). Integrative genomics reveals novel molecular pathways and gene networks for coronary artery disease. PLoS Genet..

[140] Kalousová M., Jáchymová M., Germanová A., Kubena A.A., Tesar V., Zima T. (2010). Genetic predisposition to advanced glycation end products toxicity is related to prognosis of chronic hemodialysis patients. Kidney Blood Press. Res..

[141] Kalousová M., Germanová A., Jáchymová M., Mestek O., Tesar V., Zima T. (2008). A419C (E111A) polymorphism of the glyoxalase I gene and vascular complications in chronic hemodialysis patients. Ann. N. Y. Acad. Sci..

[142] Tikellis C., Pickering R.J., Tsorotes D., Huet O., Cooper M.E., Jandeleit-Dahm K., Thomas M.C. (2014). Dicarbonyl stress in the absence of hyperglycemia increases endothelial inflammation and atherogenesis similar to that observed in diabetes. Diabetes.

[143] Blackburn N.J., Vulesevic B., Ahmadi A., McNeill B., Milne R.W., Suuronen E.J. (2013). Abstract 14257: Glyoxalase-1 Over-expression Preserves Cardiac Function Post-MI by Enhancing Vascularity and Reducing AGE Accumulation and Cardiomyocyte Apoptosis. Circulation.

[144] Ceradini D.J., Yao D., Grogan R.H., Callaghan M.J., Edelstein D., Brownlee M., Gurtner G.C. (2008). Decreasing intracellular superoxide corrects defective ischemia-induced new vessel formation in diabetic mice. J. Biol. Chem..

[145] Angeloni C., Zambonin L., Hrelia S. (2014). Role of methylglyoxal in Alzheimer’s disease. BioMed Res. Int..

[146] Ciavardelli D., Silvestri E., Del Viscovo A., Bomba M., De Gregorio D., Moreno M., Di Ilio C., Goglia F., Canzoniero L.M., Sensi S.L. (2010). Alterations of brain and cerebellar proteomes linked to Aβ and tau pathology in a female triple-transgenic murine model of Alzheimer’s disease. Cell Death Dis..

[147] Kurz A., Rabbani N., Walter M., Bonin M., Thornalley P., Auburger G., Gispert S. (2011). Alpha-synuclein deficiency leads to increased glyoxalase I expression and glycation stress. Cell. Mol. Life Sci. CMLS.

[148] Williams R.t., Lim J.E., Harr B., Wing C., Walters R., Distler M.G., Teschke M., Wu C., Wiltshire T., Su A.I. (2009). A common and unstable copy number variant is associated with differences in Glo1 expression and anxiety-like behavior. PLoS ONE.

[149] Kollmannsberger L.K., Gassen N.C., Bultmann A., Hartmann J., Weber P., Schmidt M.V., Rein T. (2013). Increased glyoxalase-1 levels in Fkbp5 knockout mice caused by glyoxalase-1 gene duplication. G3.

[150] Hovatta I., Tennant R.S., Helton R., Marr R.A., Singer O., Redwine J.M., Ellison J.A., Schadt E.E., Verma I.M., Lockhart D.J. (2005). Glyoxalase 1 and glutathione reductase 1 regulate anxiety in mice. Nature.

[151] Krömer S.A., Kessler M.S., Milfay D., Birg I.N., Bunck M., Czibere L., Panhuysen M., Pütz B., Deussing J.M., Holsboer F. (2005). Identification of glyoxalase-I as a protein marker in a mouse model of extremes in trait anxiety. J. Neurosci..

[152] Fujimoto M., Uchida S., Watanuki T., Wakabayashi Y., Otsuki K., Matsubara T., Suetsugi M., Funato H., Watanabe Y. (2008). Reduced expression of glyoxalase-1 mRNA in mood disorder patients. Neurosci. Lett..

[153] Szczepanik J.C., de Almeida G.R.L., Cunha M.P., Dafre A.L. (2020). Repeated Methylglyoxal Treatment Depletes Dopamine in the Prefrontal Cortex, and Causes Memory Impairment and Depressive-Like Behavior in Mice. Neurochem. Res..

[154] Li H., Zheng L., Chen C., Liu X., Zhang W. (2019). Brain Senescence Caused by Elevated Levels of Reactive Metabolite Methylglyoxal on D-Galactose-Induced Aging Mice. Front. Neurosci..

[155] Thornalley P.J., Rabbani N. (2011). Glyoxalase in tumourigenesis and multidrug resistance. Semin. Cell Dev. Biol..

[156] Wong K.K., deLeeuw R.J., Dosanjh N.S., Kimm L.R., Cheng Z., Horsman D.E., MacAulay C., Ng R.T., Brown C.J., Eichler E.E. (2007). A comprehensive analysis of common copy-number variations in the human genome. Am. J. Hum. Genet..

[157] Cahan P., Li Y., Izumi M., Graubert T.A. (2009). The impact of copy number variation on local gene expression in mouse hematopoietic stem and progenitor cells. Nat. Genet..

[158] Santarius T., Bignell G.R., Greenman C.D., Widaa S., Chen L., Mahoney C.L., Butler A., Edkins S., Waris S., Thornalley P.J. (2010). GLO1-A novel amplified gene in human cancer. Genes Chromosomes Cancer.

[159] Loarca L., Sassi-Gaha S., Artlett C.M. (2013). Two α-dicarbonyls downregulate migration, invasion, and adhesion of liver cancer cells in a p53-dependent manner. Dig. Liver Dis..

[160] Antognelli C., Mezzasoma L., Fettucciari K., Talesa V.N. (2013). A novel mechanism of methylglyoxal cytotoxicity in prostate cancer cells. Int. J. Biochem. Cell Biol..

[161] Young T.W., Mei F.C., Yang G., Thompson-Lanza J.A., Liu J., Cheng X. (2004). Activation of antioxidant pathways in ras-mediated oncogenic transformation of human surface ovarian epithelial cells revealed by functional proteomics and mass spectrometry. Cancer Res..

[162] Yang Y.X., Chen Z.C., Zhang G.Y., Yi H., Xiao Z.Q. (2008). A subcelluar proteomic investigation into vincristine-resistant gastric cancer cell line. J. Cell. Biochem..

[163] Sakamoto H., Mashima T., Kizaki A., Dan S., Hashimoto Y., Naito M., Tsuruo T. (2000). Glyoxalase I is involved in resistance of human leukemia cells to antitumor agent-induced apoptosis. Blood.

[164] Sakamoto H., Mashima T., Sato S., Hashimoto Y., Yamori T., Tsuruo T. (2001). Selective activation of apoptosis program by S-p-bromobenzylglutathione cyclopentyl diester in glyoxalase I-overexpressing human lung cancer cells. Clin. Cancer Res..

[165] Stratmann B., Goldstein B., Thornalley P.J., Rabbani N., Tschoepe D. (2017). Intracellular Accumulation of Methylglyoxal by Glyoxalase 1 Knock Down Alters Collagen Homoeostasis in L6 Myoblasts. Int. J. Mol. Sci..

[166] Nigro C., Leone A., Fiory F., Prevenzano I., Nicolò A., Mirra P., Beguinot F., Miele C. (2019). Dicarbonyl Stress at the Crossroads of Healthy and Unhealthy Aging. Cells.

[167] Shafie A., Xue M., Barker G., Zehnder D., Thornalley P.J., Rabbani N. (2016). Reappraisal of putative glyoxalase 1-deficient mouse and dicarbonyl stress on embryonic stem cells in vitro. Biochem. J..

[168] Jang S., Kwon D.M., Kwon K., Park C. (2017). Generation and characterization of mouse knockout for glyoxalase 1. Biochem. Biophys. Res. Commun..

[169] Moraru A., Wiederstein J., Pfaff D., Fleming T., Miller A.K., Nawroth P., Teleman A.A. (2018). Elevated Levels of the Reactive Metabolite Methylglyoxal Recapitulate Progression of Type 2 Diabetes. Cell Metab..

[170] Vulesevic B., McNeill B., Giacco F., Maeda K., Blackburn N.J., Brownlee M., Milne R.W., Suuronen E.J. (2016). Methylglyoxal-Induced Endothelial Cell Loss and Inflammation Contribute to the Development of Diabetic Cardiomyopathy. Diabetes.

[171] Li H., O’Meara M., Zhang X., Zhang K., Seyoum B., Yi Z., Kaufman R.J., Monks T.J., Wang J.M. (2019). Ameliorating Methylglyoxal-Induced Progenitor Cell Dysfunction for Tissue Repair in Diabetes. Diabetes.

[172] Qian S., Qian Y., Huo D., Wang S., Qian Q. (2019). Tanshinone IIa protects retinal endothelial cells against mitochondrial fission induced by methylglyoxal through glyoxalase 1. Eur. J. Pharmacol..

[173] Qi W., Keenan H.A., Li Q., Ishikado A., Kannt A., Sadowski T., Yorek M.A., Wu I.H., Lockhart S., Coppey L.J. (2017). Pyruvate kinase M2 activation may protect against the progression of diabetic glomerular pathology and mitochondrial dysfunction. Nat. Med..

[174] He Y., Zhou C., Huang M., Tang C., Liu X., Yue Y., Diao Q., Zheng Z., Liu D. (2020). Glyoxalase system: A systematic review of its biological activity, related-diseases, screening methods and small molecule regulators. Biomed. Pharmacother..

[175] Liu Y.W., Liu X.L., Kong L., Zhang M.Y., Chen Y.J., Zhu X., Hao Y.C. (2019). Neuroprotection of quercetin on central neurons against chronic high glucose through enhancement of Nrf2/ARE/glyoxalase-1 pathway mediated by phosphorylation regulation. Biomed. Pharmacother..

[176] Angeloni C., Turroni S., Bianchi L., Fabbri D., Motori E., Malaguti M., Leoncini E., Maraldi T., Bini L., Brigidi P. (2013). Novel targets of sulforaphane in primary cardiomyocytes identified by proteomic analysis. PLoS ONE.

[177] Santel T., Pflug G., Hemdan N.Y., Schäfer A., Hollenbach M., Buchold M., Hintersdorf A., Lindner I., Otto A., Bigl M. (2008). Curcumin inhibits glyoxalase 1: A possible link to its anti-inflammatory and anti-tumor activity. PLoS ONE.

[178] Takasawa R., Takahashi S., Saeki K., Sunaga S., Yoshimori A., Tanuma S. (2008). Structure-activity relationship of human GLO I inhibitory natural flavonoids and their growth inhibitory effects. Bioorganic Med. Chem..

[179] Qudjani E., Iman M., Davood A., Ramandi M.F., Shafiee A. (2016). Design and Synthesis of Curcumin-Like Diarylpentanoid Analogues as Potential Anticancer Agents. Recent Pat. Anti-Cancer Drug Discov..

[180] Zhang H., Zhai J., Zhang L., Li C., Zhao Y., Chen Y., Li Q., Hu X.P. (2016). In Vitro Inhibition of Glyoxalase I by Flavonoids: New Insights from Crystallographic Analysis. Curr. Top. Med. Chem..

[181] Kumar R., Bhan Tiku A. (2020). Naringenin Suppresses Chemically Induced Skin Cancer in Two-Stage Skin Carcinogenesis Mouse Model. Nutr. Cancer.

[182] Farkhondeh T., Folgado S.L., Pourbagher-Shahri A.M., Ashrafizadeh M., Samarghandian S. (2020). The therapeutic effect of resveratrol: Focusing on the Nrf2 signaling pathway. Biomed. Pharmacother..

[183] Cheng A.S., Cheng Y.H., Chiou C.H., Chang T.L. (2012). Resveratrol upregulates Nrf2 expression to attenuate methylglyoxal-induced insulin resistance in Hep G2 cells. J. Agric. Food Chem..

[184] Antognelli C., Moretti S., Frosini R., Puxeddu E., Sidoni A., Talesa V.N. (2019). Methylglyoxal Acts as a Tumor-Promoting Factor in Anaplastic Thyroid Cancer. Cells.

[185] Santini S.J., Cordone V., Mijit M., Bignotti V., Aimola P., Dolo V., Falone S., Amicarelli F. (2019). SIRT1-Dependent Upregulation of Antiglycative Defense in HUVECs Is Essential for Resveratrol Protection against High Glucose Stress. Antioxidants.

[186] Cha S.H., Hwang Y., Heo S.J., Jun H.S. (2018). Diphlorethohydroxycarmalol Attenuates Methylglyoxal-Induced Oxidative Stress and Advanced Glycation End Product Formation in Human Kidney Cells. Oxidative Med. Cell. Longev..

[187] Ávila F., Theoduloz C., López-Alarcón C., Dorta E., Schmeda-Hirschmann G. (2017). Cytoprotective Mechanisms Mediated by Polyphenols from Chilean Native Berries against Free Radical-Induced Damage on AGS Cells. Oxidative Med. Cell. Longev..

[188] Habtemariam S. (2019). Natural Products in Alzheimer’s Disease Therapy: Would Old Therapeutic Approaches Fix the Broken Promise of Modern Medicines?. Molecules.

[189] Arbo B.D., André-Miral C., Nasre-Nasser R.G., Schimith L.E., Santos M.G., Costa-Silva D., Muccillo-Baisch A.L., Hort M.A. (2020). Resveratrol Derivatives as Potential Treatments for Alzheimer’s and Parkinson’s Disease. Front. Aging Neurosci..

[190] Suantawee T., Thilavech T., Cheng H., Adisakwattana S. (2020). Cyanidin Attenuates Methylglyoxal-Induced Oxidative Stress and Apoptosis in INS-1 Pancreatic β-Cells by Increasing Glyoxalase-1 Activity. Nutrients.

[191] Frandsen J.R., Narayanasamy P. (2018). Neuroprotection through flavonoid: Enhancement of the glyoxalase pathway. Redox Biol..

[192] Maher P., Dargusch R., Ehren J.L., Okada S., Sharma K., Schubert D. (2011). Fisetin lowers methylglyoxal dependent protein glycation and limits the complications of diabetes. PLoS ONE.

[193] Frandsen J., Narayanasamy P. (2017). Flavonoid Enhances the Glyoxalase Pathway in Cerebellar Neurons to Retain Cellular Functions. Sci. Rep..

[194] Zhu X., Cheng Y.Q., Lu Q., Du L., Yin X.X., Liu Y.W. (2018). Enhancement of glyoxalase 1, a polyfunctional defense enzyme, by quercetin in the brain in streptozotocin-induced diabetic rats. Naunyn-Schmiedeberg’s Arch. Pharmacol..

[195] Van den Eynde M.D.G., Geleijnse J.M., Scheijen J., Hanssen N.M.J., Dower J.I., Afman L.A., Stehouwer C.D.A., Hollman P.C.H., Schalkwijk C.G. (2018). Quercetin, but Not Epicatechin, Decreases Plasma Concentrations of Methylglyoxal in Adults in a Randomized, Double-Blind, Placebo-Controlled, Crossover Trial with Pure Flavonoids. J. Nutr..

[196] Frandsen J., Choi S.R., Narayanasamy P. (2020). Neural Glyoxalase Pathway Enhancement by Morin Derivatives in an Alzheimer’s Disease Model. ACS Chem. Neurosci..

[197] Chen Y.J., Kong L., Tang Z.Z., Zhang Y.M., Liu Y., Wang T.Y., Liu Y.W. (2019). Hesperetin ameliorates diabetic nephropathy in rats by activating Nrf2/ARE/glyoxalase 1 pathway. Biomed. Pharmacother..

[198] Xue M., Weickert M.O., Qureshi S., Kandala N.B., Anwar A., Waldron M., Shafie A., Messenger D., Fowler M., Jenkins G. (2016). Improved Glycemic Control and Vascular Function in Overweight and Obese Subjects by Glyoxalase 1 Inducer Formulation. Diabetes.

[199] Irshad Z., Xue M., Ashour A., Larkin J.R., Thornalley P.J., Rabbani N. (2019). Activation of the unfolded protein response in high glucose treated endothelial cells is mediated by methylglyoxal. Sci. Rep..

[200] Zhao Y., Wang P., Sang S. (2019). Dietary Genistein Inhibits Methylglyoxal-Induced Advanced Glycation End Product Formation in Mice Fed a High-Fat Diet. J. Nutr..

[201] Suh K.S., Chon S., Choi E.M. (2018). Cytoprotective effects of xanthohumol against methylglyoxal-induced cytotoxicity in MC3T3-E1 osteoblastic cells. J. Appl. Toxicol..

[202] Huang X., Wang J., Chen X., Liu P., Wang S., Song F., Zhang Z., Zhu F., Huang X., Liu J. (2018). The Prenylflavonoid Xanthohumol Reduces Alzheimer-Like Changes and Modulates Multiple Pathogenic Molecular Pathways in the Neuro2a/APP(swe) Cell Model of AD. Front. Pharmacol..

[203] Liu Y.W., Zhu X., Yang Q.Q., Lu Q., Wang J.Y., Li H.P., Wei Y.Q., Yin J.L., Yin X.X. (2013). Suppression of methylglyoxal hyperactivity by mangiferin can prevent diabetes-associated cognitive decline in rats. Psychopharmacology.

[204] Liu Y.W., Cheng Y.Q., Liu X.L., Hao Y.C., Li Y., Zhu X., Zhang F., Yin X.X. (2017). Mangiferin Upregulates Glyoxalase 1 Through Activation of Nrf2/ARE Signaling in Central Neurons Cultured with High Glucose. Mol. Neurobiol..

[205] Liu Y.W., Zhu X., Zhang L., Lu Q., Wang J.Y., Zhang F., Guo H., Yin J.L., Yin X.X. (2013). Up-regulation of glyoxalase 1 by mangiferin prevents diabetic nephropathy progression in streptozotocin-induced diabetic rats. Eur. J. Pharmacol..

[206] Nagaraj R.H., Sarkar P., Mally A., Biemel K.M., Lederer M.O., Padayatti P.S. (2002). Effect of pyridoxamine on chemical modification of proteins by carbonyls in diabetic rats: Characterization of a major product from the reaction of pyridoxamine and methylglyoxal. Arch. Biochem. Biophys..

[207] Wetzels S., Wouters K., Miyata T., Scheijen J., Hendriks J.J.A., Schalkwijk C.G., Vanmierlo T. (2018). Advanced Glycation Endproducts Are Increased in the Animal Model of Multiple Sclerosis but Cannot Be Reduced by Pyridoxamine Treatment or Glyoxalase 1 Overexpression. Int. J. Mol. Sci..

[208] Abouzed T.K., Munesue S., Harashima A., Masuo Y., Kato Y., Khailo K., Yamamoto H., Yamamoto Y. (2016). Preventive Effect of Salicylate and Pyridoxamine on Diabetic Nephropathy. J. Diabetes Res..

[209] Oh S., Ahn H., Park H., Lee J.I., Park K.Y., Hwang D., Lee S., Son K.H., Byun K. (2019). The attenuating effects of pyridoxamine on adipocyte hypertrophy and inflammation differ by adipocyte location. J. Nutr. Biochem..

[210] Kuricova K., Pleskacova A., Pacal L., Kankova K. (2016). 1,25-Dihydroxyvitamin D increases the gene expression of enzymes protecting from glucolipotoxicity in peripheral blood mononuclear cells and human primary endothelial cells. Food Funct..

[211] Omidian M., Djalali M., Javanbakht M.H., Eshraghian M.R., Abshirini M., Omidian P., Alvandi E., Mahmoudi M. (2019). Effects of vitamin D supplementation on advanced glycation end products signaling pathway in T2DM patients: A randomized, placebo-controlled, double blind clinical trial. Diabetol. Metab. Syndr..

[212] Matsuo K., Watanabe T., Takenaka A. (2019). Effect of dietary vitamin E on oxidative stress-related gene-mediated differences in anxiety-like behavior in inbred strains of mice. Physiol. Behav..

[213] Derakhshanian H., Djalali M., Mohammad Hassan M.H., Alvandi E., Eshraghian M.R., Mirshafiey A., Nadimi H., Jahanabadi S., Zarei M., Djazayery A. (2019). Vitamin D suppresses cellular pathways of diabetes complication in liver. Iran. J. Basic Med Sci..

[214] Wang Z.H., Hsu C.C., Huang C.N., Yin M.C. (2010). Anti-glycative effects of oleanolic acid and ursolic acid in kidney of diabetic mice. Eur. J. Pharmacol..

[215] Hsu W.H., Lee B.H., Chang Y.Y., Hsu Y.W., Pan T.M. (2013). A novel natural Nrf2 activator with PPARγ-agonist (monascin) attenuates the toxicity of methylglyoxal and hyperglycemia. Toxicol. Appl. Pharmacol..

[216] Truong C.S., Seo E., Jun H.S. (2019). Psoralea corylifolia L. Seed Extract Attenuates Methylglyoxal-Induced Insulin Resistance by Inhibition of Advanced Glycation End Product Formation. Oxidative Med. Cell. Longev..

[217] Cha S.H., Hwang Y., Heo S.J., Jun H.S. (2019). Indole-4-carboxaldehyde Isolated from Seaweed, Sargassum thunbergii, Attenuates Methylglyoxal-Induced Hepatic Inflammation. Mar. Drugs.

[218] Wang Z., Zhang J., Chen L., Li J., Zhang H., Guo X. (2019). Glycine Suppresses AGE/RAGE Signaling Pathway and Subsequent Oxidative Stress by Restoring Glo1 Function in the Aorta of Diabetic Rats and in HUVECs. Oxidative Med. Cell. Longev..

[219] Wang Z., Zhao D., Chen L., Li J., Yuan G., Yang G., Zhang H., Guo X., Zhang J. (2019). Glycine increases glyoxalase-1 function by promoting nuclear factor erythroid 2-related factor 2 translocation into the nucleus of kidney cells of streptozotocin-induced diabetic rats. J. Diabetes Investig..

