# Supplementary material for: The Glyoxalase System in Age-Related Diseases: Nutritional Intervention as Anti-Ageing Strategy
Source: Cells. 2021 Jul 22;10(8):1852. doi: 10.3390/cells10081852 (PMC8393707; doi:10.3390/cells10081852)
Supplement: Supplementary file 1 [file cells-10-01852-s001.zip › cells-1259927-supplementary.pdf]

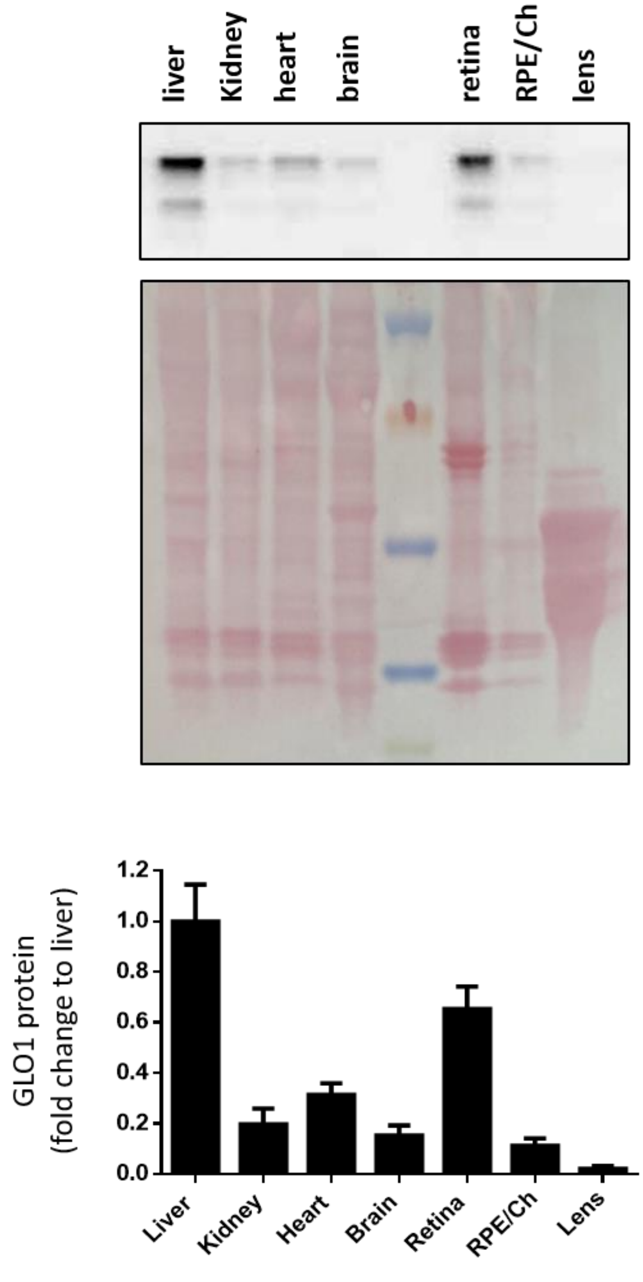

Figure S1. Differential expression of GLO1 in mouse tissues. (**A**) Representative western blot analysis of non-ocular (left) and ocular (right) tissue extracts (50 ug/lane) using a polyclonal antibody for GLO1 (commercial, GeneTex) and (**B**) protein quantification of GLO1 expressed as fold change to liver (*n* = 3). Data shown are mean ± standard errors of the means (SEM).
